# Tolerability of [^161^Tb]Tb-SibuDAB in comparison to other PSMA-targeting radioligands in the preclinical setting

**DOI:** 10.1007/s00259-026-08129-1

**Published:** 2026-08-13

**Authors:** Korbinian Krieger, Ana Katrina Mapanao, Julie Bolcaen, Kristel Kegler, Peter Bernhardt, Nicholas P. van der Meulen, Ulli Köster, Alin Chirindel, Guillaume P. Nicolas, Roger Schibli, Cristina Müller

**Affiliations:** 1 Department of Chemistry and Applied Biosciences, ETH Zurich, Zurich, Switzerland; 2 PSI Center for Life Sciences, Villigen‑PSI, Switzerland; 3 AnaPath Services Ltd, Liestal, Switzerland; 4 Department of Medical Radiation Sciences, Institution of Clinical Science, Sahlgrenska Academy, University of Gothenburg, Gothenburg, Sweden; 5 Department of Medical Physics and Biomedical Engineering (MFT), Sahlgrenska University Hospital, Gothenburg, Sweden; 6 PSI Center for Nuclear Engineering and Sciences, Villigen‑PSI, Switzerland; 7 Institut Laue-Langevin, Grenoble, France; 8 Division of Nuclear Medicine, University Hospital Basel, Basel, Switzerland

**Keywords:** Radioligand therapy, Prostate cancer, PSMA, Terbium-161, Albumin-binder

## Abstract

**Purpose:**

The successful preclinical efficacy studies prompted a Phase I clinical trial to investigate [^161^Tb]Tb-SibuDAB. Deeper insight into potential undesired effects on normal tissues and organs remained to be investigated. The aim of this preclinical study was, therefore, to assess and compare the tolerability of [^161^Tb]Tb-SibuDAB with that of [^177^Lu]Lu-SibuDAB, [^161^Tb]Tb-PSMA-I&T and [^177^Lu]Lu-PSMA-I&T.

**Methods:**

The time-dependent tissue distribution profiles of [^161^Tb]Tb-SibuDAB and [^161^Tb]Tb-PSMA-I&T were assessed in immunocompetent mice for dosimetry estimations. Substructural activity distribution in kidneys was further investigated ex vivo. In Study I, [^161^Tb]Tb-SibuDAB, [^177^Lu]Lu-SibuDAB, [^161^Tb]Tb-PSMA-I&T and [^177^Lu]Lu-PSMA-I&T were administered at 30 MBq/mouse while in Study II, additional activities of 15 MBq and 60 MBq [^161^Tb]Tb-SibuDAB were applied. Blood cell counts were determined on Days 10, 28 and 56 after radioligand injection while blood and bone marrow smears, blood plasma biomarkers and selected organs were investigated at study end on Day 56.

**Results:**

Organ uptake and absorbed doses were severalfold higher for SibuDAB than for PSMA-I&T and terbium-161 delivered about 40% higher organ doses than lutetium-177. Kidney accumulation of radioligands was mainly localized in the renal cortex and blockable with excess ligand in the case of PSMA-I&T but not for SibuDAB. In Study I, leukocyte and erythrocyte levels remained comparable between treated and control mice. Thrombocyte counts remained stable with PSMA-I&T radioligands, while SibuDAB radioligands caused 23–28% decrease by Day 10, with full recovery seen for [^161^Tb]Tb-SibuDAB. In Study II, 60 MBq [^161^Tb]Tb-SibuDAB did not significantly reduce leukocyte levels, but erythrocyte counts were marginally lower on Days 10 and 28. Thrombocytes decreased to 18%, 23% and 44% of control values on Day 10 after application of 15 MBq, 30 MBq and 60 MBq, respectively. Thrombocyte counts recovered over time, although average values in some groups remained modestly decreased on Day 56. In both studies, blood plasma biomarkers were similar between treated and control groups. Histological assessment of bone marrow, spleen, liver and kidney showed only minimal alterations (score ≤ 1/3).

**Conclusion:**

Hematological changes were transient, but more pronounced after the application of SibuDAB than of PSMA-I&T, most likely due to the prolonged blood circulation. Other organs and tissues remained unaffected by any of the applied treatments. Substituting lutetium-177 with terbium-161 did not significantly alter the measured parameters. It remains to be demonstrated clinically whether ^161^Tb-based radioligands can be administered at activity levels comparable to their ^177^Lu-based analogues.

**Graphical abstract:**

**Figure:**
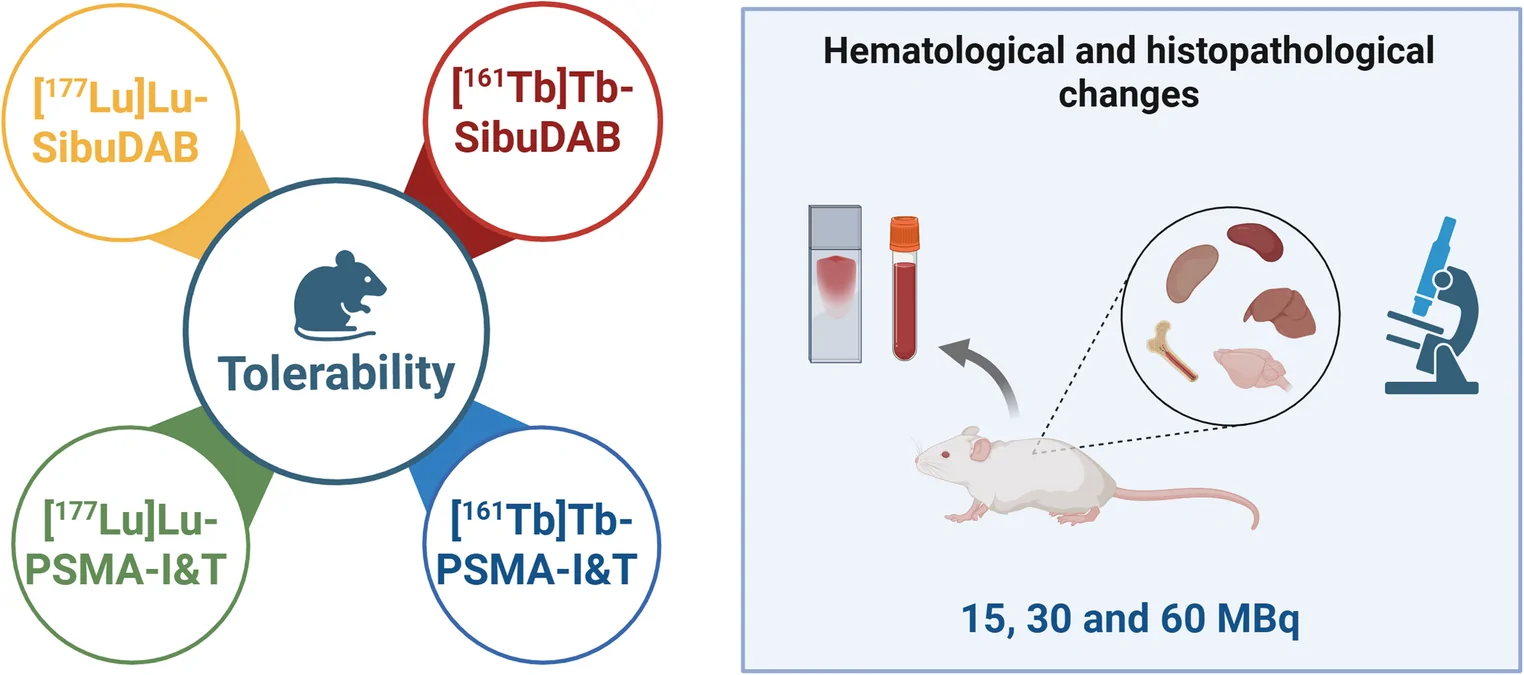

**Supplementary Information:**

The online version contains supplementary material available at https://doi.org/10.1007/s00259-026-08129-1.

## Introduction

Radioligand therapy (RLT) targeting the prostate-specific membrane antigen (PSMA) is an established treatment modality for metastatic castration-resistant prostate cancer (mCRPC), primarily utilizing [^177^Lu]Lu-PSMA-617 (Pluvicto™, Novartis Pharma Switzerland AG) [1]) and [^177^Lu]Lu-PSMA-I&T [2]. In the Phase III VISION trial, [^177^Lu]Lu-PSMA-617 was well tolerated and extended the median overall survival of patients in the post-taxane setting from 11.3 to 15.3 months [3]. While this novel treatment modality provides significant initial benefit in patients with mCRPC, disease recurrence and progression ultimately remain unavoidable [4]. In recent years, radionuclides with more favorable decay characteristics have been proposed to enhance the efficacy of PSMA-targeted RLT, among those also terbium-161. This radionuclide has attracted the interest of researchers and clinicians due to its similarity to lutetium-177 and its co-emission of short-ranged electrons (conversion and Auger electrons), thereby enhancing the potential for their effective eradication [5–7]. The superior efficacy of terbium-161 over lutetium-177 to treat tumors in mice has been demonstrated in multiple preclinical studies with a variety of targeting agents, including PSMA-617 and PSMA-I&T [8, 9]. Only few preclinical studies focused on potential increased risk of damage to normal tissues associated with terbium-161’s emission of short-ranged electrons [10, 11].

In clinics, the safety profile and therapeutic potential of [^161^Tb]Tb-PSMA-617 have been reported as favorable in individual patients [12–14]. Furthermore, initial results from the Phase I/II trial (VIOLET; NCT05521412) indicate the promising efficacy of [^161^Tb]Tb-PSMA-I&T for the treatment of mCRPC patients [15, 16]. At the Paul Scherrer Institute (PSI), we developed albumin-binding PSMA radioligands to prolong blood circulation and thereby enhance tumor uptake [17–20]. Among these, SibuDAB [20], a PSMA ligand modified with (*S*)-ibuprofen, was evaluated preclinically in combination with terbium-161 and lutetium-177 to assess and compare the therapeutic potential of the corresponding radioligands [9]. In this study, SibuDAB inhibited the tumor growth of PC-3 PIP tumor-bearing mice more effectively than PSMA-I&T, and the ^161^Tb-labeled version outperformed the ^177^Lu-based analogue. An initial dosimetry study of [^177^Lu]Lu-SibuDAB in mCRPC patients revealed an almost 2-fold higher absorbed dose to tumors than reported for conventional PSMA radioligands, while tumor-to-organ dose ratios were similar [21]. In a cohort of 7 patients, up to four cycles of [^177^Lu]Lu-SibuDAB were well tolerated with positive treatment responses while mild myelotoxicity was observed in a minority of patients with baseline hematopoietic impairment [22]. The positive outcome of these studies and the preclinical data [9] motivated the clinical translation of [^161^Tb]Tb-SibuDAB, which is currently being tested in a Phase Ia/b trial at University Hospital Basel in Switzerland (PROGNOSTICS; NCT06343038) [23].

The prolonged blood circulation of SibuDAB leads to an increased absorbed dose to the bone marrow but also other organs and tissues which warrants further investigation into the consequent tolerability profile of [^161^Tb]Tb-SibuDAB. The aim of this study was, therefore, to compare effects on blood cells as a result of bone marrow impairment as well as effects on other organs and tissues following administration of SibuDAB and PSMA-I&T labeled with either terbium-161 or lutetium-177.

## Materials and methods

### Radioligand preparation

**Fig. 1:**
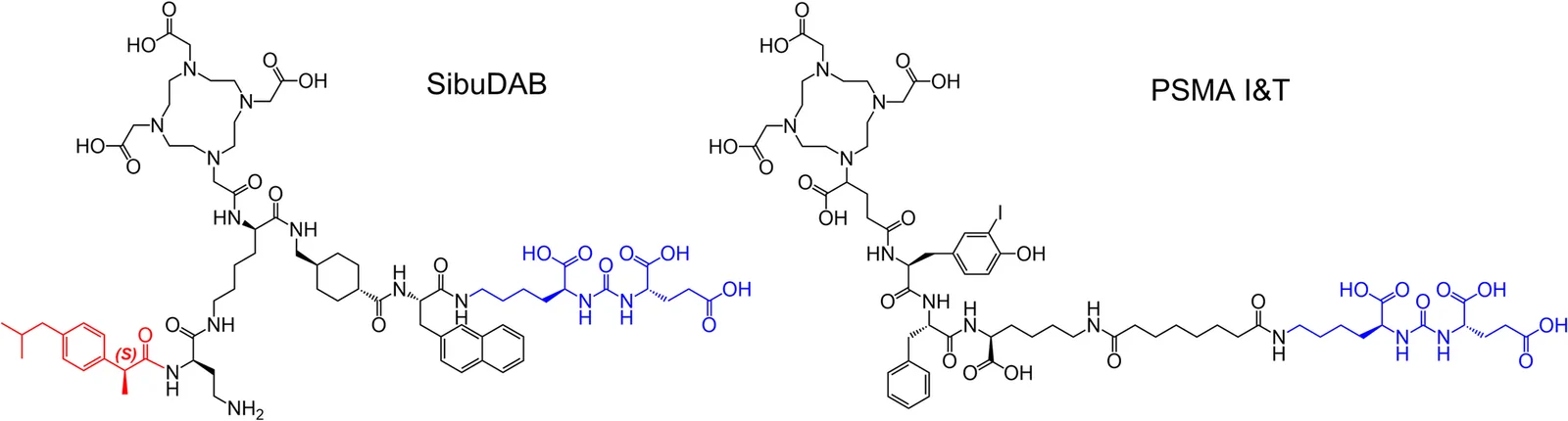
Chemical structures of SibuDAB and PSMA-I&T, composed of the PSMA targeting entity (blue), a macrocyclic chelator (DOTA or DOTAGA, respectively) and, in the case of SibuDAB, an albumin binder based on (*S*)-ibuprofen (red)

SibuDAB (*S*-diastereoisomer of Ibu-DAB-PSMA [20]) and PSMA-I&T were labeled with terbium-161 [24] and lutetium-177 under standard labeling conditions at molar activity of 5–60 MBq/nmol (Fig. 1) [9]. The radioligands were obtained with a radiochemical purity of > 95% and used without further purification steps (Supplementary Material, Fig. S1).

### *In vivo* studies

All applicable international, national and/or institutional guidelines for the care and use of laboratory animals were followed. The experiments were performed following the Swiss regulations for animal welfare and approved by the cantonal committee of animal experimentation (License N° 75668). Immunocompetent female FVB mice (FVB/NCrl) were purchased from Charles River Laboratories (Sulzfeld, Germany). Upon arrival at PSI, the mice were acclimatized for at least 7 days before performing the studies. Female mice were used as they can be housed in groups over extended periods without the risk of fighting and related injuries which is commonly observed in male mice.

### Biodistribution studies

Mice were intravenously injected with [^161^Tb]Tb-SibuDAB or [^161^Tb]Tb-PSMA-I&T (5 MBq, 1 nmol) and sacrificed 1 h, 4 h, 24 h and 72 h later. A ligand amount of 1 nmol per mouse was used, consistent with previous preclinical studies [9, 18]. This amount corresponded to approximately double the ligand mass typically administered in patients (~ 200 µg) when accounting for differences in body mass and surface area while extrapolating values from mice to men [25]. Organs and tissues were collected, weighed and measured for activity using a γ-counter (Wallac Wizard 1480, PerkinElmer, Waltham, MA, USA). Decay-corrected values of the injected activity per tissue mass (% IA/g) were calculated as the average ± standard deviation (SD) of *n* = 3 mice per timepoint (Supplementary Material).

### Dosimetry estimation

The absorbed doses to the kidneys, spleen, liver, lungs, salivary glands and femur containing the bone marrow were estimated using the non-decay-corrected biodistribution data of [^161^Tb]Tb-SibuDAB and [^161^Tb]Tb-PSMA-I&T. The accumulated activity was plotted against time and the bi-exponential curve fits were integrated to infinite to obtain the time-integrated activity concentration (TIAC). Dosimetry calculations were performed using the Moby mouse phantom as model [26]. The equal tissue distribution profiles of the ^161^Tb-labeled PSMA ligands and their corresponding ^177^Lu-labeled counterparts were previously confirmed [9]. Dose estimations for [^177^Lu]Lu-SibuDAB and [^177^Lu]Lu-PSMA-I&T were, thus, based on the tissue distribution data of their respective ^161^Tb-based counterparts, while taking the difference in absorbed energy and physical half-life between terbium-161 and lutetium-177 into account.

### *Ex vivo* SPECT imaging and autoradiography of kidneys

Mice were injected with [^177^Lu]Lu-SibuDAB or [^177^Lu]Lu-PSMA-I&T (20 MBq, 1 nmol) without or with pre-injected excess of the respective ligand (50 nmol) to block PSMA. At 4 h after injection, the kidneys were collected to measure the mass and accumulated activity. One kidney was used for ex vivo SPECT/CT imaging (NanoSPECT/CT, Mediso Medical Imaging Systems, Budapest, Hungary) and the second kidney was embedded in Tissue-Tek, frozen and cut into sections for ex vivo autoradiography using a phosphor screen (Super resolution screen Cat. No. 7001487, PerkinElmer, Waltham MA, USA) and a storage phosphor system (Cyclone Plus, PerkinElmer, Waltham MA, USA) (Supplementary Material).

### Design of the tolerability studies in mice

**Fig. 2:**
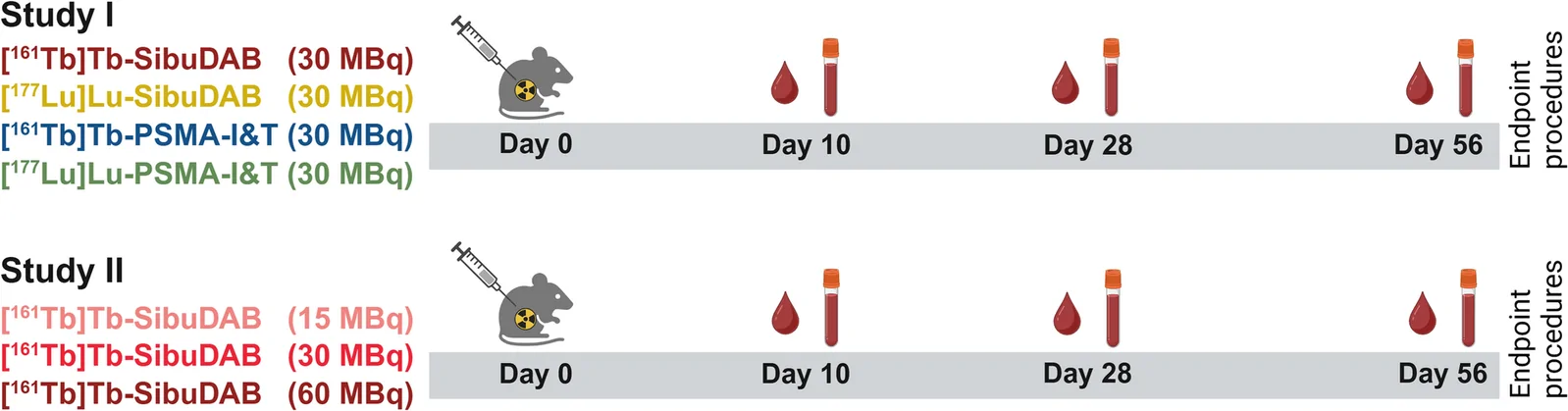
Design of the studies to compare the tolerability of the four PSMA radioligands applied at same amount of activity (30 MBq; Study I) or investigate the effects of increasing activity levels of [^161^Tb]Tb-SibuDAB (15 MBq, 30 MBq and 60 MBq; Study II)

The tolerability of the PSMA radioligands was assessed in immunocompetent mice without tumors to evaluate effects on normal tissues only at predefined endpoints. Power calculations using G*power software (version 3.1.9.7) [27] were based on the findings of Busslinger et al. regarding tolerability of somatostatin-based radiopeptides in similar settings (Supplementary Material) [11]. The Studies were designed as follows (Fig. 2): In Study I, the tolerability of [^161^Tb]Tb-SibuDAB (*n* = 6) was compared to that of [^177^Lu]Lu-SibuDAB (*n* = 5; *n* = 1 mouse was excluded early during the study), as well as that of [^161^Tb]Tb-PSMA-I&T (*n* = 6) and [^177^Lu]Lu-PSMA-I&T (*n* = 6). Mice were injected with 30 MBq (1 nmol) radioligand, which would correspond to approximately 6.8 GBq in a human patient following a body mass and surface area-based extrapolation conceptualized by Nair et al. (Supplementary Material) [25]. In Study II, [^161^Tb]Tb-SibuDAB was applied at 15 MBq, 30 MBq (same group as in Study I) or 60 MBq per mouse (*n* = 6 per group), which would translate to approximately 3.4 GBq, 6.8 GBq and 14 GBq, respectively, in humans. Control mice were injected with vehicle only (0.05% bovine serum albumin in 0.9% saline).

### Monitoring of the mice in study I and study II

After radioligand application, the mice were monitored for the following 56 days. The body mass of the mice was recorded three times a week and expressed relative to the body mass on the day of injection (Day 0; set as 1.0). Peripheral blood was collected on Days 10, 28 and 56 to determine blood cell counts using a hematology analyzer (VetScan HM5, Abaxis, Union City, CA, USA). At study end on Day 56, blood plasma biomarkers including blood urea nitrogen, alkaline phosphatase and albumin were determined using a dry chemistry analyzer (DRI-CHEM 4000i, FUJIFILM, Tokyo, Japan). Blood smears from the peripheral blood taken on Day 56, as well as bone marrow smears, were prepared (Fig. 2). The brain, spleen, kidneys and liver were collected, weighed and fixed in formaldehyde (4%) for further analyses (Supplementary Material).

### Histopathological analysis

Blood and bone marrow smears were stained using the Pappenheim method [28]. The 4-µm thick paraffin sections of the spleen, liver and kidneys were stained using hematoxylin and eosin and the histo(patho)logical analysis was performed by a veterinary pathologist using a pre-defined scoring system (Supplementary Material) [11].

### Statistical analysis

Blood cell counts were analyzed using two-way ANOVA, whereas all other data were analyzed using one-way ANOVA. The Dunnett’s multiple comparison post-test was applied to compare the results of treated groups to that of the control group. The Tukey’s multiple comparison post-test was employed to compare the outcome among different groups of mice treated with the radioligands (GraphPad Prism, version 10.5.0). The software-generated *p*-values are automatically adjusted for multiple comparisons and *p* < 0.05 was considered statistically significant.

## Results

### Biodistribution study

**Fig. 3:**
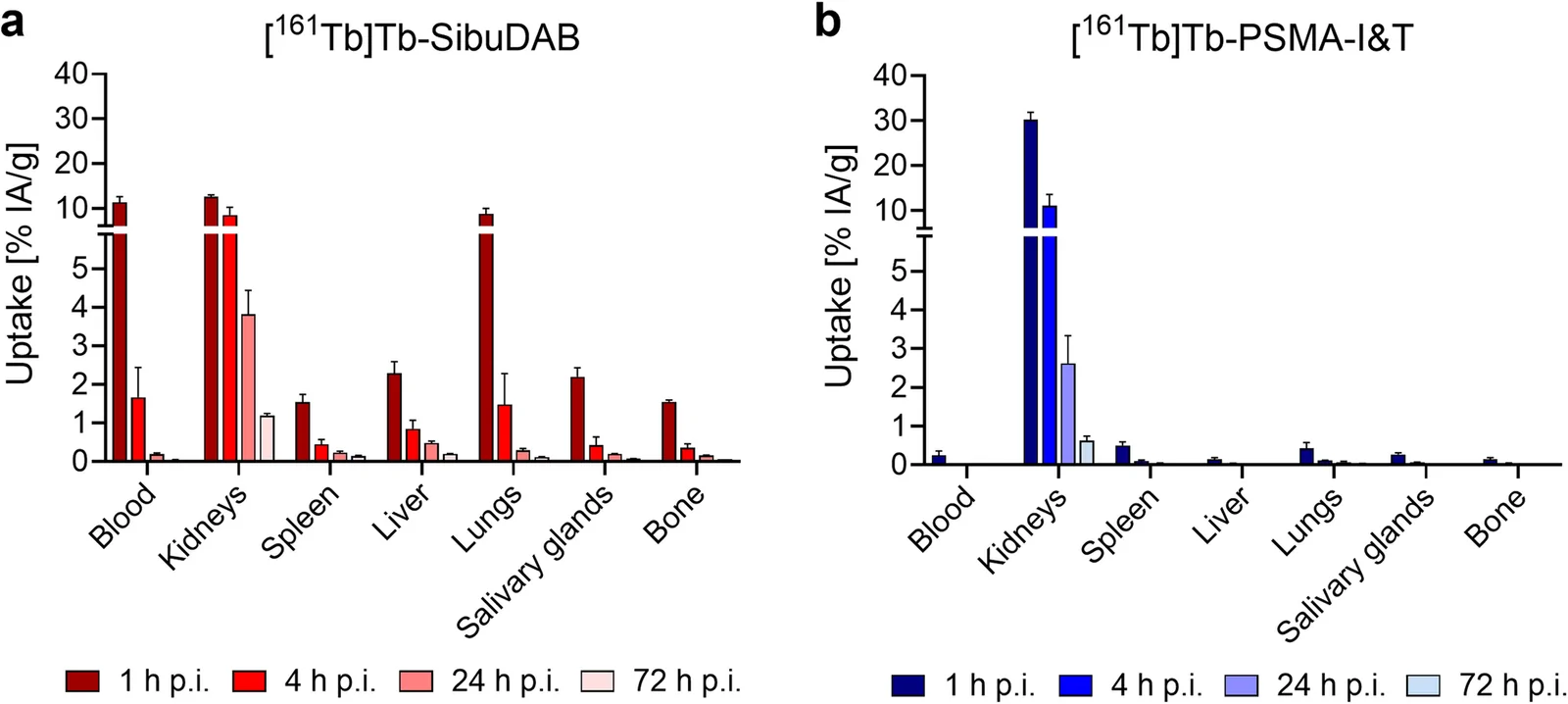
**a/b** Tissue distribution data of the PSMA radioligands determined in immunocompetent FVB mice. **a** Biodistribution data of [^161^Tb]Tb-SibuDAB; **b** Biodistribution data of [^161^Tb]Tb-PSMA-I&T. Data are shown as average ± SD (*n* = 3); p.i.: post-injection

At 1 h after injection of [^161^Tb]Tb-SibuDAB, blood activity reached 11 ± 1% IA/g, but it was cleared effectively over time to 1.7 ± 0.8% IA/g at 4 h after injection and < 0.2% IA/g at 24 h after injection. Renal uptake of [^161^Tb]Tb-SibuDAB was ~ 13% IA/g at 1 h and 3.8 ± 0.6% IA/g at 24 h after injection. In the lungs, the retention of activity was 8.8 ± 1.3% IA/g at 1 h after injection, with almost identical clearance as seen in the blood. At the same timepoint, uptake of [^161^Tb]Tb-SibuDAB in the liver was 2.3 ± 0.3% IA/g but cleared to < 0.5% IA/g at 24 h after injection. In other relevant organs and tissues such as the spleen, salivary glands and bones, the activity retention was ≤ 2.2% IA/g at 1 h after injection and declined further thereafter (Fig. 3a; Supplementary Material, Table S1).

Blood activity after injection of [^161^Tb]Tb-PSMA-I&T was < 0.5% IA/g already after 1 h and declined further to < 0.2% IA/g at the 24-h timepoint. While renal uptake 1 h after injection of [^161^Tb]Tb-PSMA-I&T was 30 ± 2% IA/g, only 11 ± 3% IA/g and 2.6 ± 0.7% IA/g were measured at 4 h and 24 h after injection, respectively. In other organs and tissues including liver, spleen, lungs, salivary glands and bones, the residual activity was ≤ 0.5% IA/g already 1 h after injection of [^161^Tb]Tb-PSMA-I&T (Fig. 3b; Supplementary Material, Table S2).

### Dosimetry estimation

The estimated absorbed organ dose to the kidneys was similar for [^161^Tb]Tb-SibuDAB and [^161^Tb]Tb-PSMA-I&T. Absorbed doses to the spleen, liver and lungs were 4.5-, 12- and 7.5-fold higher for [^161^Tb]Tb-SibuDAB than for [^161^Tb]Tb-PSMA-I&T, respectively. The absorbed dose to the salivary glands was 7.2-fold higher for [^161^Tb]Tb-SibuDAB than for [^161^Tb]Tb-PSMA-I&T. In the femur, which includes the bone marrow, the absorbed dose was 5.4-fold higher for [^161^Tb]Tb-SibuDAB than for [^161^Tb]Tb-PSMA-I&T. Assuming equal tissue distribution irrespective of the employed radionuclide, the absorbed organ doses for the respective ^177^Lu-based analogues were approximately 1.4-fold lower (Table 1).

**Table 1:** Absorbed organ doses^†^

Organ	[^161^Tb]Tb-SibuDAB	[^177^Lu]Lu-SibuDAB	[^161^Tb]Tb-PSMA-I&T	[^177^Lu]Lu-PSMA-I&T
	[mGy/MBq]	[mGy/MBq]	[mGy/MBq]	[mGy/MBq]
Kidneys	302 ± 21	215 ± 15	275 ± 28	198 ± 19
Spleen	27 ± 3	19 ± 3	6.0 ± 0.7	4.4 ± 0.8
Liver	44 ± 2	32 ± 1	3.7 ± 0.3	2.5 ± 0.2
Lungs	38 ± 3	27 ± 2	5.1 ± 0.6	3.7 ± 0.3
Salivary glands	19 ± 1	14 ± 1	2.7 ± 0.2	1.9 ± 0.2
Femur^‡^	13.3 ± 0.2	9.4 ± 0.2	2.4 ± 0.2	1.7 ± 0.1

^†^Calculated based on non-decay-corrected biodistribution data obtained for [^161^Tb]Tb-SibuDAB and [^161^Tb]Tb-PSMA-I&T and extrapolated to the respective ^177^Lu-labeled counterparts. ^‡^Femur containing bone marrow

### *Ex vivo* SPECT imaging and autoradiography of kidneys

**Fig. 4:**
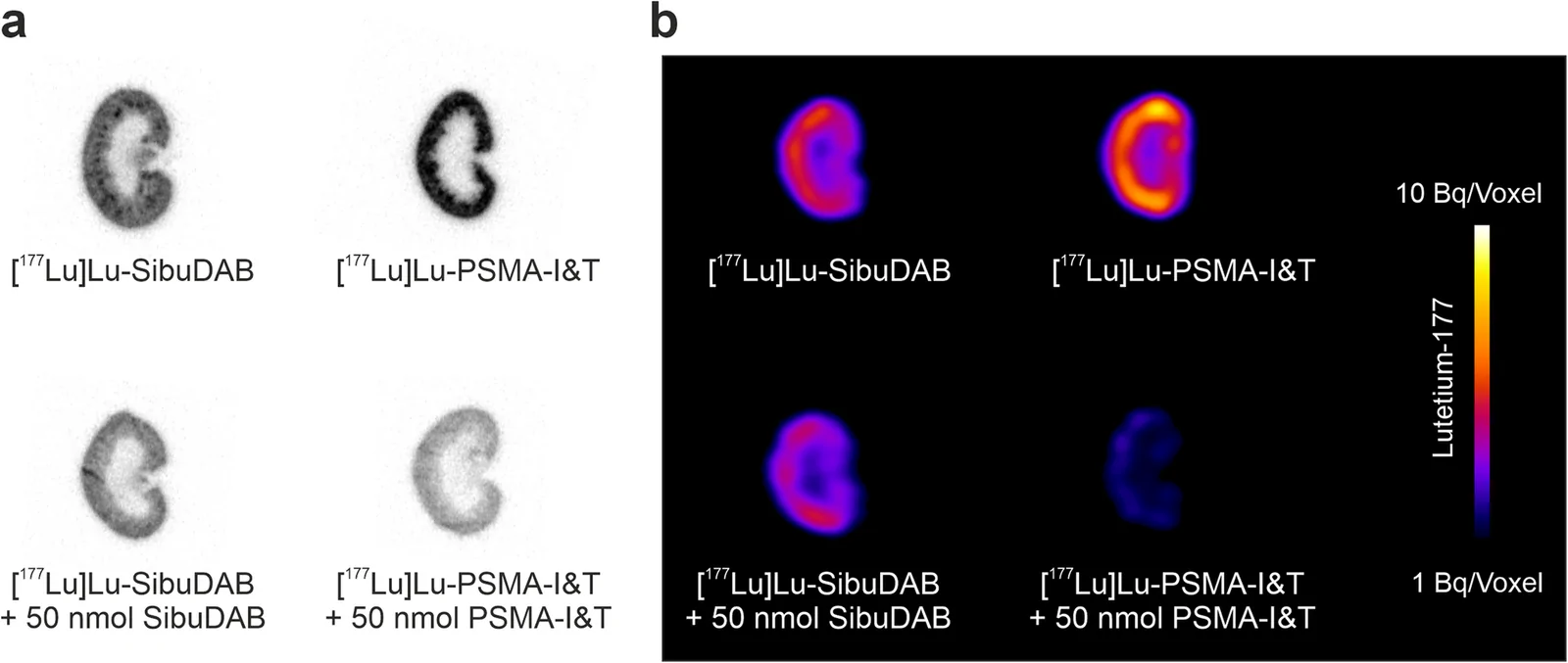
**a/b** Renal accumulation at 4 h after injection of [^177^Lu]Lu-SibuDAB or [^177^Lu]Lu-PSMA-I&T in absence or presence of excess unlabeled ligand. **a** Ex vivo autoradiographs; **b** Ex vivo SPECT images

At 4 h after injection, ex vivo SPECT imaging and ex vivo autoradiography of frozen kidney sections showed uptake of [^177^Lu]Lu-PSMA-I&T and [^177^Lu]Lu-SibuDAB in the renal cortex. Pre-injection of excess unlabeled PSMA-I&T effectively reduced renal uptake of [^177^Lu]Lu-PSMA-I&T to 22% of the value obtained after injection of [^177^Lu]Lu-PSMA-I&T only. Excess unlabeled SibuDAB did, however, not substantially affect the uptake of [^177^Lu]Lu-SibuDAB, which remained at 83% of the value for the uptake in mice injected with the radioligand only (Fig. 4; Supplementary Material).

### Study I: tolerability of ^161^Tb- and ^177^Lu-labeled PSMA radioligands (30 MBq/mouse)

Similar to the mice of the control group, the animals injected with 30 MBq of ^161^Tb- and ^177^Lu-labeled SibuDAB and PSMA-I&T gained body mass over time (Supplementary Material, Fig. S2). One mouse that received [^177^Lu]Lu-SibuDAB had to be euthanized on Day 10 due to body mass loss exceeding 15%, in accordance with the predefined endpoint criteria. As no apparent treatment-related cause was identified upon histopathological investigation, this event was assumed unrelated to the applied radioligand.

**Fig. 5:**
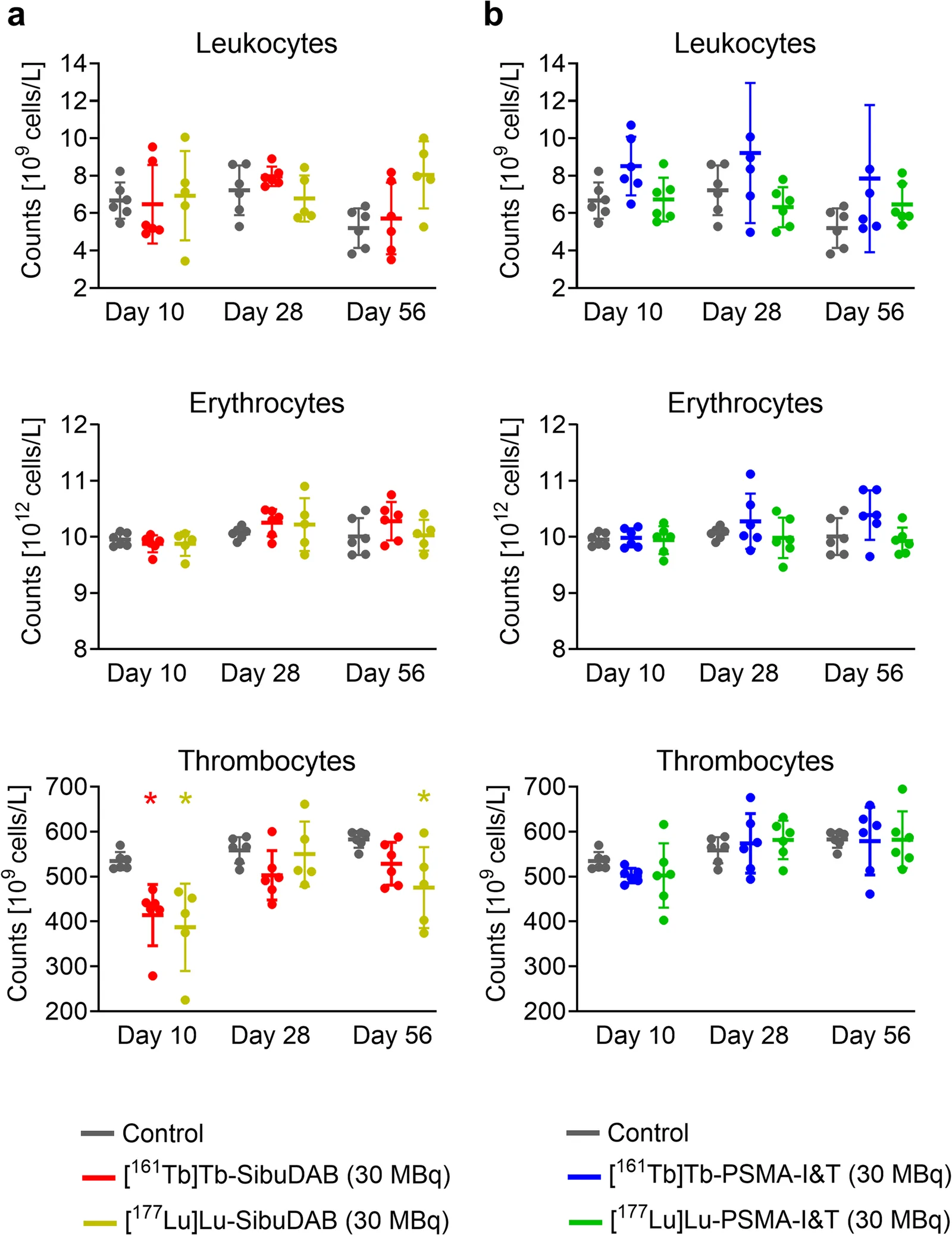
**a/b** Leukocyte, erythrocyte and thrombocyte counts of mice injected with the respective radioligand measured on Days 10, 28 and 56. **a** [^161^Tb]Tb-SibuDAB and [^177^Lu]Lu-SibuDAB (30 MBq per mouse); **b** [^161^Tb]Tb-PSMA-I&T and [^177^Lu]Lu-PSMA-I&T (30 MBq per mouse). *Values that differed significantly from those obtained from the control group (*p* < 0.05)

On Day 10, the average leukocyte counts of mice injected with 30 MBq of [^161^Tb]Tb-SibuDAB, [^177^Lu]Lu-SibuDAB or [^177^Lu]Lu-PSMA-I&T were between 6.5 × 10^9^ and 6.9 × 10^9^ cells/L, which was comparable to (6.7 ± 1.0)×10^9^ cells/L measured from the control group (*p* > 0.05). At the same timepoint, mice injected with 30 MBq [^161^Tb]Tb-PSMA-I&T had 27% higher leukocyte counts ((8.5 ± 1.6)×10^9^ cells/L) than the control animals (*p* > 0.05). On Day 28, no statistically significant changes in leukocyte counts were determined between treated mice and untreated controls (*p* > 0.05). Treated mice had, however, higher leukocyte counts on Day 56, although this difference was statistically not significant (*p* > 0.05; Fig. 5; Supplementary Material, Table S3). Erythrocyte counts were unaffected by the treatment with radioligands and remained constant between 9.9 × 10^12^ and 10.4 × 10^12^ cells/L at all investigated timepoints (Fig. 5; Supplementary Material, Table S3). On Day 10, thrombocyte counts in mice injected with [^161^Tb]Tb-PSMA-I&T and [^177^Lu]Lu-PSMA-I&T were similar to that of the control mice (*p* > 0.05), however, mice that received [^161^Tb]Tb-SibuDAB or [^177^Lu]Lu-SibuDAB had 23–28% lower average thrombocyte counts (*p* < 0.05) with no statistically significant difference between these two groups (*p* > 0.05). Mice injected with [^177^Lu]Lu-SibuDAB showed significantly fewer thrombocytes on Day 10 ((387 ± 97)×10^9^ cells/L) than those injected with [^161^Tb]Tb-PSMA-I&T or [^177^Lu]Lu-PSMA-I&T ((502–503) × 10^9^ cells/L; *p* < 0.05). On Day 28, the average thrombocyte counts from mice of the control and treated groups were between 503 × 10^9^ cells/L and 582 × 10^9^ cells/L (*p* > 0.05). The average thrombocyte values obtained on Day 56 after application of [^161^Tb]Tb-SibuDAB, [^161^Tb]Tb-PSMA-I&T or [^177^Lu]Lu-PSMA-I&T ranged from 529 × 10^9^ cells/L to 582 × 10^9^ cells/L and were comparable to those of control mice (*p* > 0.05). Mice injected with [^177^Lu]Lu-SibuDAB had an average thrombocyte counts of (476 ± 90)×10^9^ cells/L, which was significantly lower than that of the control group (*p* < 0.05). (Fig. 5; Supplementary Material, Table S3).

No significant differences in the blood plasma concentrations of blood urea nitrogen, alkaline phosphatase and albumin were determined between treated mice and untreated controls on Day 56 (Supplementary Material, Table S4). Similarly, the masses of the brain, kidneys, liver and spleen were comparable for treated mice and untreated controls and similar among the different treatment groups. The same held true for the respective organ-to-brain mass ratios (Supplementary Material, Table S5).

### Histopathological findings

Peripheral blood smears prepared on Day 56 showed increased presence of immature granulocytes (metamyelocytes) in all treated mice (*n* = 23/23). Immature precursors of neutrophils (band neutrophils) were also increased in treated mice compared to untreated controls and were observed more often in mice that received ^161^Tb-based radioligands (75%; *n* = 9/12) than those injected with ^177^Lu-based radioligands (55%; *n* = 6/11). Other blood precursor cells, such as myeloblast and metarubricytes, were not seen in the samples from mice injected with [^161^Tb]Tb-SibuDAB but present in 2–3 mice of other groups that received other radioligands (Supplementary Material, Table S6). Based on the bone marrow smears, the average myeloid-to-erythroid ratios in treated mice were 1.51–1.86, which was insignificantly higher than the 1.22 ± 0.37 noted for the control group (*p* > 0.05; Supplementary Material, Table S7).

**Fig. 6:**
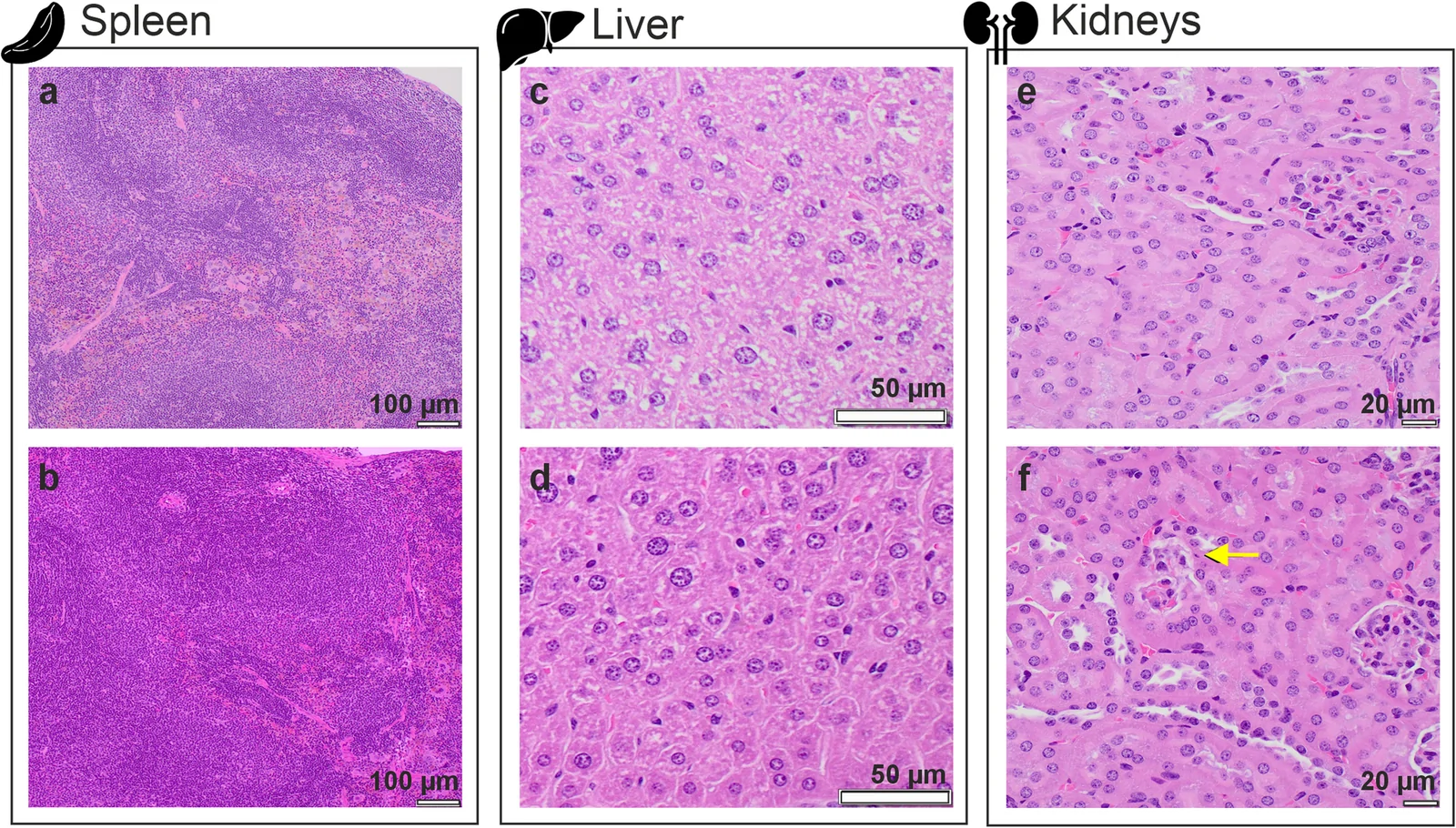
**a–f** Representative images of treatment-related histopathological findings. **a** Spleen tissue from a control mouse; **b** Spleen tissue from a mouse treated with 30 MBq [^161^Tb]Tb-SibuDAB showing minimal lymphoid hyperplasia (darker violet color). **c** Liver tissue of a control mouse; **d** Liver tissue of a mouse treated with 60 MBq [^161^Tb]Tb-SibuDAB with hepatocellular hypertrophy. **e** Kidney tissue of a control mouse; **f** Kidney tissue of a mouse treated with 30 MBq [^177^Lu]Lu-PSMA-I&T showing minimal tubular degeneration (yellow arrow)

Histopathological analyses of the spleen, kidneys and liver were performed using a pre-defined scoring system (Supplementary Material, Table S8). Minimal splenic lymphoid hyperplasia was seen in 83% (*n* = 5/6) of mice treated with [^161^Tb]Tb-SibuDAB and in 1–2 mice injected with the other radioligands but absent (*n* = 0/6) in the control mice (Fig. 6a/b; Supplementary Material, Fig. S3). Minimal hepatocellular hypertrophy was observed in 13% (*n* = 3/23) of the radioligand-injected mice, while absent in the control group (Fig. 6c/d; Supplementary Material, Fig. S3). Minimal renal tubular degeneration was present only in 17% (*n* = 1/6) of the mice treated with [^177^Lu]Lu-PSMA-I&T, whereas minimal renal tubular basophilia was also observed in only 17% (*n* = 1/6) of the mice treated with [^161^Tb]Tb-SibuDAB, [^177^Lu]Lu-PSMA-I&T and [^161^Tb]Tb-PSMA-I&T (Fig. 6e/f; Supplementary Material, Fig. S3).

### Study II: tolerability of [^161^Tb]Tb-SibuDAB applied at variable activities

**Fig. 7:**
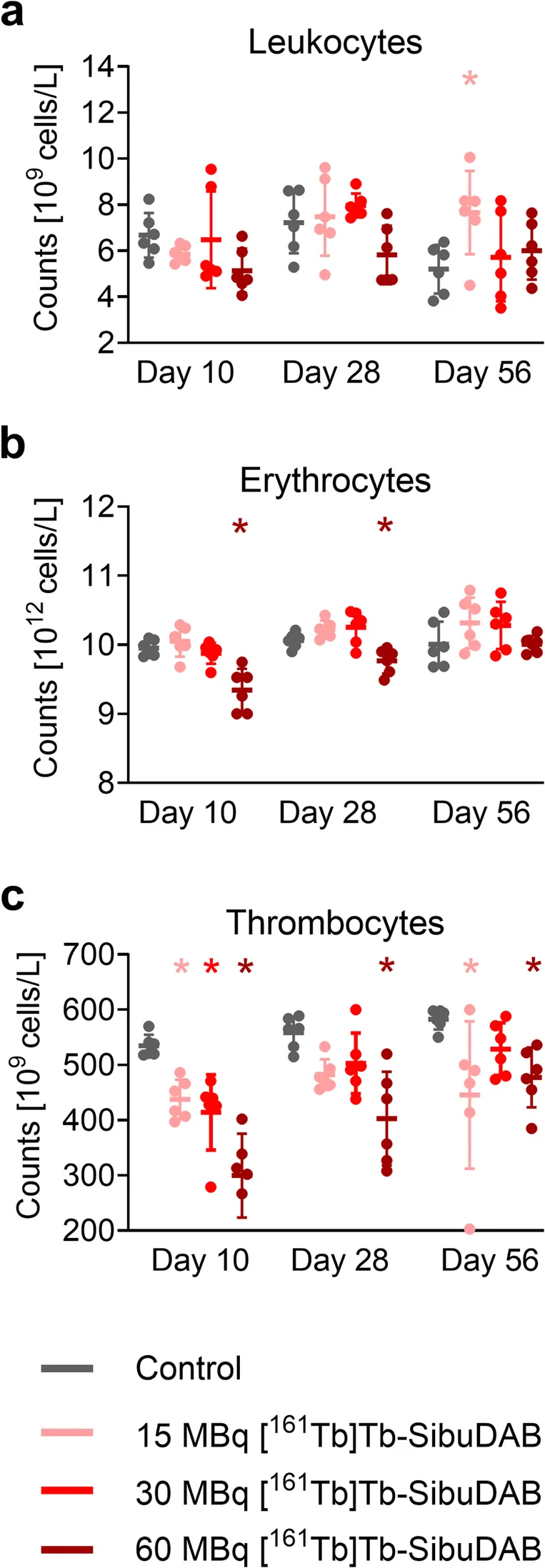
**a–c** Changes in blood cell counts of mice treated with 15 MBq, 30 MBq or 60 MBq of [^161^Tb]Tb-SibuDAB. **a** Leukocyte counts; **b** erythrocyte counts; **c** thrombocyte counts. *Values that differed significantly from those obtained from the control group (*p* < 0.05)

The body mass of mice injected with [^161^Tb]Tb-SibuDAB increased over the study duration, regardless of the quantity of injected activity and was comparable to the trend observed in untreated controls (Supplementary Material, Fig. S4). Leukocyte counts of the control and treated mice were within the same range, except on Day 56 when higher average values were measured for mice injected with 15 MBq [^161^Tb]Tb-SibuDAB (*p* < 0.05; Fig. 7a; Supplementary Material, Table S9). Erythrocyte counts of control mice and those injected with 15 MBq or 30 MBq of [^161^Tb]Tb-SibuDAB were in the range of (9.9–10.1)×10^12^ cells/L on Day 10, whereas mice treated with 60 MBq [^161^Tb]Tb-SibuDAB had significantly lower average erythrocyte counts of (9.3 ± 0.3)×10^12^ cells/L (*p* < 0.05). While the mice treated with 60 MBq [^161^Tb]Tb-SibuDAB still had significantly fewer erythrocytes on Day 28 (*p* < 0.05), the values normalized by Day 56 when the mice of all groups had erythrocyte counts between 10.0 × 10^12^ cells/L and 10.3 × 10^12^ cells/L (Fig. 7b; Supplementary Material, Table S9). Average thrombocyte counts in all mice injected with [^161^Tb]Tb-SibuDAB were between 300 × 10^9^ and 438 × 10^9^ cells/L on Day 10, which was significantly lower than the counts of (535 ± 20)×10^9^ cells/L measured for the control group (*p* < 0.05). In addition, mice treated with 60 MBq [^161^Tb]Tb-SibuDAB had significantly fewer thrombocytes than those that received 15 MBq or 30 MBq (*p* < 0.05). The thrombocyte counts in mice injected with 15 MBq or 30 MBq of [^161^Tb]Tb-SibuDAB improved on Day 28 ((483–503)×10^9^ cells/L), while mice treated with 60 MBq radioligand had an average value of (403 ± 85)×10^9^ cells/L which remained significantly lower than that of the control group ((559 ± 29)×10^9^ cells/L; *p* < 0.05). On Day 56, the average thrombocyte count in mice treated 60 MBq [^161^Tb]Tb-SibuDAB was (478 ± 54)×10^9^ cells/L, which was significantly lower than that of the control animals ((583 ± 19)×10^9^ cells/L; *p* < 0.05). Mice that received 30 MBq radioligand instead reached an average value of (529 ± 48)×10^9^ cells/L, which was not significantly different from the control animals (*p* > 0.05) (Fig. 7c; Supplementary Material, Table S9).

Mice treated with [^161^Tb]Tb-SibuDAB showed lower blood urea nitrogen concentrations, but it was only significant for the group that received 60 MBq (8.4 ± 1.6 mM vs. 12 ± 3 mM in control mice; *p* < 0.05). Concentrations of alkaline phosphatase and albumin in blood plasma of control and treated mice were in the same range (*p* > 0.05; Supplementary Material, Table S10).

Brain, kidneys, liver and spleen mass, as well as the corresponding organ-to-brain mass ratios of mice treated with 15 MBq and 30 MBq of [^161^Tb]Tb-SibuDAB were comparable to that of the control group (*p* > 0.05). Mice treated with 60 MBq [^161^Tb]Tb-SibuDAB had comparable brain, liver and spleen mass and organ-to-brain ratios, but the kidneys mass and respective kidney-to-brain mass ratio were significantly higher than the values obtained for untreated controls (*p* < 0.05; Supplementary Material, Table S11).

### Histopathological findings

Increased metamyelocytes and band neutrophils were observed in blood smears of 94% (*n* = 17/18) and 67% (*n* = 12/18) of mice injected with [^161^Tb]Tb-SibuDAB, respectively, with no correlation of the incidence to the amount of injected activity (Supplementary Material, Table S12). The average myeloid-to-erythroid ratios from the treated mice ranged from 1.39 to 1.64 and were slightly higher than the 1.22 ± 0.37 observed in control mice (*p* > 0.05; Supplementary Material, Table S13). While absent in control animals, signs of minimal splenic lymphoid hyperplasia were seen in 56% (*n* = 10/18) of the treated mice but no correlation between the incidence and the quantity of injected activity was seen (Supplementary Material, Fig. S5). Hepatocellular hypertrophy was noted in 33% (*n* = 2/6) of mice treated with 30 MBq or 60 MBq [^161^Tb]Tb-SibuDAB but was not observed in the control mice and those that received 15 MBq (Supplementary Material, Fig. S5). Minimal renal tubular degeneration and basophilia were identified in only few treated mice and the incidence did not correlate to the quantity of activity applied (Supplementary Material, Fig. S5).

## Discussion

Biodistribution data from immunocompetent mice demonstrated longer blood retention of [^161^Tb]Tb-SibuDAB than of [^161^Tb]Tb-PSMA-I&T due to the albumin-binding properties of SibuDAB. This was consistent with previously reported data from tumor-bearing nude mice [9]. As a result, the absorbed dose to the bone marrow, which was inferred from the marrow-containing femur, revealed an up to 7.8- and 5.5-fold higher value after administration of [^161^Tb]Tb-SibuDAB and [^177^Lu]Lu-SibuDAB than when using the clinically established [^177^Lu]Lu-PSMA-I&T. This aligns with initial data obtained in patients, where the bone marrow was identified as the dose-limiting organ for [^177^Lu]Lu-SibuDAB [21, 22]. Nevertheless, the analysis of the bone marrow smears revealed only minimal changes in treated mice compared to untreated controls, irrespective of the applied radioligand. The absence of abnormalities in precursor cells of the erythroid and myeloid lineages further suggested that the observed hematological changes would be reversible.

Blood cell counts were determined on Days 10, 28 and 56 after radioligand application, as previously done for other radioligands [11, 29]. Changes in blood cell counts were minimal up to an applied activity of 30 MBq radioligand per mouse. This aligned with studies of other groups that reported marginal and reversible hematological changes in mice injected with 120–200 MBq [^177^Lu]Lu-PSMA-617 [30, 31]. Furthermore, our data showed that substituting lutetium-177 with terbium-161 did not significantly alter the leukocyte, erythrocyte and thrombocyte counts at any of the investigated timepoints in mice treated with PSMA-I&T or SibuDAB. A drop in thrombocyte counts was observed after application of [^177^Lu]Lu-SibuDAB and [^161^Tb]Tb-SibuDAB, while this was not the case when mice received [^177^Lu]Lu-PSMA-I&T or [^161^Tb]Tb-PSMA-I&T. These findings were expected as a result of the longer blood residence time of SibuDAB, in line with previous findings that showed more pronounced hematological changes for albumin-binding PSMA radioligands (e.g. [^177^Lu]Lu-PSMA-ALB-56) than for conventional ones [32]. At the end of Study II, mice that received 60 MBq [^161^Tb]Tb-SibuDAB showed thrombocyte levels that were ~ 18% lower than controls, however, with the nadir reached on Day 10, thrombocyte recovery was clearly in progress. Restoration of blood cell counts was also influenced by compensatory mechanisms as indicated by the presence of immature erythrocytes in peripheral blood and minimal lymphoid hyperplasia in the spleen, an active hematopoietic site in mice [33]. Even milder effects on blood cell counts would be expected in a therapeutic setting, as the quantity of activity used in the present study exceeded the necessary activity to treat PC-3 PIP tumor in mice [9]. In a recent single-center Phase I/II trial, 7.4 GBq [^161^Tb]Tb-PSMA-I&T showed a favorable safety profile in mCRPC patients with only few cases of grade 3 lymphocytopenia that were related to the treatment [16]. Hematological effects after treatment with ~ 6.8 GBq [^161^Tb]Tb-PSMA-617 were found to be minimal as well [34]. Initial data from a Phase I trial with [^161^Tb]Tb-SibuDAB showed higher tumor and bone marrow absorbed dose [23], however, efficacy and safety aspects need to be further investigated.

Renal accumulation of [^161^Tb]Tb-SibuDAB was comparable to [^161^Tb]Tb-PSMA-I&T, consistent with previous findings in PC-3 PIP tumor-bearing mice [9]. It was reported in literature that [^177^Lu]Lu-PSMA-617 was cleared more efficiently from kidneys than [^177^Lu]Lu-PSMA-I&T in mice [35] although this difference was less pronounced in human patients [36, 37]. It is unclear whether the DOTAGA chelator of PSMA-I&T was responsible for these observations [38]. The estimated renal dose of patients that received [^177^Lu]Lu-SibuDAB was considerably higher than for the conventional PSMA radioligands [21].

In our study, the histological findings for the mouse kidneys were minimal and largely consistent with background lesions irrespective of the radioligand administered. Although renal damage in mice is likely to occur 6–8 months after treatment for absorbed dose above 23–30 Gy [10], long term nephrotoxicity was not expected with the investigated PSMA radioligands as the highest estimated dose was ~ 18 Gy with 60 MBq [^161^Tb]Tb-SibuDAB. The accumulation of SibuDAB and PSMA-I&T in the renal cortex is expected to result in a heterogeneous renal dose distribution, presumably leading to substantially higher absorbed doses at the nephron level for terbium-161 than for lutetium-177, as recently reported for [^161^Tb]Tb-DOTATATE and [^177^Lu]Lu-DOTATATE [39]. As SibuDAB uptake was only marginally reduced by pre-injection of excess ligand, uptake mechanisms beyond PSMA-mediated binding likely contribute to its renal accumulation. This observation may provide an opportunity to apply previously established strategies to reduce kidneys retention of radiopharmaceuticals [40].

In other mouse organs, including the lungs, liver and salivary glands, the retention of SibuDAB was higher than that of PSMA-I&T which, again, can be ascribed to the enhanced blood circulation time of the former. These findings aligned with previous data obtained in tumor-bearing mice [9] and with clinical observations [21]. No indication of severe treatment-related hepatotoxicity was observed in mice, regardless of the applied radioligand. The higher absorbed dose to the spleen for SibuDAB than for PSMA-I&T may have contributed to the lymphoid hyperplasia observed in mice injected with [^161^Tb]Tb‑SibuDAB. Although the absorbed dose to the salivary glands was increased in mice, data in patients showed improved tumor-to-salivary gland ratios with [^177^Lu]Lu-SibuDAB compared to conventional PSMA radioligands [21].

The therapeutic efficacy and associated side effects of the herein reported radioligands were previously assessed in tumor-bearing mice [9]. In the present study, we used immunocompetent mice without tumor to enable longitudinal comparisons at defined timepoints without confounding effects from tumor sink, variable timepoints of endpoint-related euthanasia or immune deficiency. Female mice were employed due to practical considerations, although the use of male mice would, in principle, be more appropriate for testing radioligands for prostate cancer. Important is the fact that mice appear to be less susceptible to radiation effects than human patients [30]. Therefore, the reported data should be interpreted primarily as a comparative assessment of the PSMA radioligands and not be considered directly transferable to the clinical situation. Clearly, more clinical data regarding the efficacy and safety of ^161^Tb-based radiopharmaceuticals in patients are to be awaited.

## Conclusion

The data of these preclinical studies indicate that [^161^Tb]Tb-SibuDAB is well tolerated, with adverse events comparable to those observed for [^177^Lu]Lu-SibuDAB and for ^161^Tb- and ^177^Lu-based PSMA-I&T at a single injection of 30 MBq per mouse. Effects on blood cell counts in mice were minimal and the initial decline was followed by steady recovery, even at 60 MBq [^161^Tb]Tb-SibuDAB. Terbium-161 was shown to be equally well tolerated as lutetium-177, however, the absorbed dose to normal organs and tissues was substantially higher for SibuDAB than for PSMA-I&T. This would entail careful dose-escalation studies in patients and likely adjustments to the commonly administered activity in clinical practice.

## Supplementary Information

Below is the link to the electronic supplementary material.

Supplementary Material 1 (DOCX 1.27 MB)

## Data Availability

All preclinical data analyzed in this study are included in this published article and in the Supplementary Material. Additional information or more detailed data are available from the corresponding author on reasonable request.
