# Supplementary material for: Tolerability of [161Tb]Tb-SibuDAB in comparison to other PSMA-targeting radioligands in the preclinical setting
Source: Eur J Nucl Med Mol Imaging. 2026 Aug 13;53(13):8284–96. doi: 10.1007/s00259-026-08129-1 (PMC13727872; doi:10.1007/s00259-026-08129-1)
Supplement: Supplementary file 1 [file 259_2026_8129_MOESM1_ESM.docx]

**Supplementary Material**

**Tolerability of [^161^Tb]Tb-SibuDAB in Comparison to other PSMA-targeting Radioligands in the Preclinical Setting**

Korbinian Krieger^1,2†^, Ana Katrina Mapanao^2†^, Julie Bolcaen^2^, Kristel Kegler^3^, Peter Bernhardt^4,5^, Nicholas P. van der Meulen^2,6^, Ulli Köster^7^, Alin Chirindel^8^, Guillaume P. Nicolas^8^, Roger Schibli^1,2^ and Cristina Müller^1,2*^

^†^ equally contributed

^1^ Department of Chemistry and Applied Biosciences, ETH Zurich, Zurich, Switzerland

^2^ PSI Center for Life Sciences, Villigen‑PSI, Switzerland

^3^ AnaPath Services Ltd., Liestal, Switzerland

^4^ Department of Medical Radiation Sciences, Institution of Clinical Science, Sahlgrenska Academy, University of Gothenburg, Gothenburg, Sweden

^5^ Department of Medical Physics and Biomedical Engineering (MFT), Sahlgrenska University Hospital, Gothenburg, Sweden

^6^ PSI Center for Nuclear Engineering and Sciences, Villigen‑PSI, Switzerland
^7^ Institut Laue-Langevin, Grenoble, France

^8^ Division of Nuclear Medicine, University Hospital Basel, Basel, Switzerland

***Correspondence to**:

Prof. Dr. Cristina Müller

Center for Radiopharmaceutical Sciences

PSI, Center for Life Sciences

Forschungsstrasse 111

5232 Villigen-PSI

Switzerland

e-mail: cristina.mueller@psi.ch

phone: +41 56 310 44 54; fax: +41 56 310 28 49

**1. Radioligand preparation**

***Purpose:*** The PSMA ligands were labeled with terbium-161 and lutetium-177 for the preclinical studies.

***Methods:*** A stock solution of SibuDAB (developed by Müller and co-workers and synthesized by the group [1]) was prepared in Milli-Q water to obtain a concentration of 1 mM. A 1-mM stock solution of PSMA-I&T (ABX Advanced Biochemical Compounds GmbH, Radeberg, Germany) was also prepared using Milli-Q water with 10% (v/v) dimethyl sulfoxide. Terbium-161, produced according to a previously published method [2] and formulated as no-carrier added [^161^Tb]TbCl_3_ in 0.05 M HCl was obtained from the Radionuclide Development group at PSI. In addition, terbium-161 was obtained from TerThera, The Netherlands, via Medisynt AG, (formerly Solumedics AG), Switzerland. Lutetium-177, formulated as no-carrier added [^177^Lu]LuCl_3_ in 0.04 M HCl, was obtained from ITM Medical Isotopes GmbH (Munich, Germany). The labeling of the PSMA ligands with terbium-161 or lutetium-177 was performed in a 1:5 (v/v) mixture of sodium acetate (0.5 M) and HCl (0.05 M) at pH 4.5 and incubated at 95 °C for 10 min as previously described [3]. The quality of the resultant PSMA radioligands was assessed using high performance liquid chromatography (HPLC), using a Merck Hitachi LaChrom HPLC system, equipped with a D-7000 interface, a L-7200 autosampler, a radioactivity detector (LB 506 B; Berthold) and a L-7100 pump connected with a reversed-phase C18 column (Xterra^TM^ MS, C18, 5 μm, 150 × 4.6 mm; Waters). A linear gradient of the mobile phase consisting of Milli-Q water with 0.1% (v/v) trifluoroacetic acid (95–20%) and acetonitrile (5–80%) was used over 15 min at a flow rate of 1 mL/min. A sample of the reaction mixture was diluted in Milli-Q water containing pentasodium diethylenetriaminepentaacetate (Na_5_-DTPA, 50 µM) prior to injection into HPLC.

***Results:*** The PSMA radioligands based on SibuDAB and PSMA-I&T, respectively, were obtained with >95% radiochemical purity at a molar activity of up to 60 MBq/nmol (Fig. S1). The radioligands were employed without further purification steps for the herein reported preclinical investigations.

**Fig. S1 a–d** Representative HPLC chromatograms of the PSMA radioligands prepared at 60 MBq/nmol with the retention time (t_R_) and radiochemical (RC) purity indicated in the graphics. **a** [^161^Tb]Tb-SibuDAB; **b** [^177^Lu]Lu-SibuDAB; **c** [^161^Tb]Tb-PSMA-I&T and **d** [^177^Lu]Lu-PSMA-I&T. Traces of unreacted radionuclide appeared with a retention time (t_R_) of ~2.9 min

**2. Biodistribution studies**

***Purpose:*** The tissue distribution of [^161^Tb]Tb-SibuDAB and [^161^Tb]Tb-PSMA-I&T was assessed in immunocompetent FVB mice to enable dosimetry estimations of relevant organs and tissues.

***Methods:*** Female FVB mice were intravenously injected with [^161^Tb]Tb-SibuDAB (5 MBq, 1 nmol per mouse) or [^161^Tb]Tb-PSMA-I&T (5 MBq, 1 nmol per mouse) diluted in saline (0.9% NaCl) containing 0.05% bovine serum albumin. The injected ligand amount (1 nmol/mouse) was kept consistent with previous preclinical studies performed in our group to investigate [^161^Tb]Tb-SibuDAB and [^161^Tb]Tb-PSMA-I&T in tumor-bearing mice [3]. Based on the extrapolation that considers the ratio of body masses (70/0.025) and surface areas (1/12.3) of humans and mice [4], a ligand amount of 1 nmol injected in mice with an approximate body mass of 25 g would correspond to about double of the ligand mass (~200 μg) commonly used in human patients with a body mass of about 70 kg. Mice were sacrificed at 1 h, 4 h, 24 h or 72 h post injection (p.i.) to collect selected organs and tissues. The tissue samples were weighed, followed by counting for activity at the same time as the standards, referring to 2.5%, 5% and 10% of injected activity, using a γ-counter (Wallac Wizard 1480, PerkinElmer, Waltham MA, USA). Decay-corrected percentages of injected activity per tissue mass (% IA/g) were calculated as average ± standard deviation (SD) for n=3 mice per timepoint. The biodistribution data were tabulated as decay-corrected values and used as non-decay-corrected values for the estimations of the absorbed organ doses. Previous preclinical studies demonstrated similar tissue distribution of ^161^Tb- and ^177^Lu-labeled PSMA ligands [3, 5]. Hence, the biodistribution results obtained for [^161^Tb]Tb-SibuDAB and [^161^Tb]Tb-PSMA-I&T were considered to reflect that of their respective ^177^Lu-based counterparts.

***Results:*** The results of the biodistribution studies are reported in the main article and listed in Tables S1 and S2.

**Table S1** Decay-corrected biodistribution data of [^161^Tb]Tb-SibuDAB in immunocompetent FVB mice at various time points after injection of [^161^Tb]Tb-SibuDAB. Decay-corrected data of accumulated activity are shown as % IA/g tissue, representing the average ± SD

|  | **[^161^Tb]Tb-SibuDAB** | | | |
| --- | --- | --- | --- | --- |
| **Organ** | **1 h p.i.** | **4 h p.i.** | **24 h p.i.** | **72 h p.i.** |
|  | n=3 | n=3 | n=3 | n=3 |
|  | [% IA/g] | [% IA/g] | [% IA/g] | [% IA/g] |
| Blood | 11 ± 1 | 1.7 ± 0.8 | 0.19 ± 0.03 | <0.05 |
| Heart | 4.0 ± 0.6 | 0.68 ± 0.36 | 0.16 ± 0.02 | <0.05 |
| Lung | 8.8 ± 1.3 | 1.5 ± 0.8 | 0.29 ± 0.05 | 0.12 ± 0.01 |
| Spleen | 1.5 ± 0.2 | 0.45 ± 0.12 | 0.23 ±0.04 | 0.14 ± 0.02 |
| Kidneys | 12.6 ± 0.4 | 8.5 ± 1.7 | 3.8 ± 0.6 | 1.2 ± 0.1 |
| Stomach | 1.3 ± 0.2 | 0.31 ± 0.12 | 0.12 ± 0.01 | <0.05 |
| Intestines | 1.6 ± 0.4 | 0.34 ± 0.13 | 0.10 ± 0.02 | <0.05 |
| Liver | 2.3 ± 0.3 | 0.85 ± 0.22 | 0.48 ± 0.05 | 0.20 ± 0.01 |
| Salivary glands | 2.2 ± 0.2 | 0.43 ± 0.21 | 0.20 ± 0.01 | 0.07 ± 0.01 |
| Muscle | 1.29 ± 0.03 | 0.27 ± 0.12 | 0.10 ± 0.02 | <0.05 |
| Bone | 1.6 ± 0.1 | 0.36 ± 0.10 | 0.16 ± 0.01 | 0.06 ± 0.01 |

**Table S2** Decay-corrected biodistribution data of [^161^Tb]Tb-PSMA-I&T in immunocompetent FVB mice at various time points after injection of [^161^Tb]Tb-PSMA-I&T. Decay-corrected data of accumulated activity are shown as % IA/g tissue, representing the average ± SD

|  | **[^161^Tb]Tb-PSMA-I&T** | | | |
| --- | --- | --- | --- | --- |
| **Organ** | **1 h p.i.** | **4 h p.i.** | **24 h p.i.** | **72 h p.i.** |
|  | n=3 | n=3 | n=3 | n=3 |
|  | [% IA/g] | [% IA/g] | [% IA/g] | [% IA/g] |
| Blood | 0.25 ± 0.11 | <0.05 | <0.05 | <0.05 |
| Heart | 0.21 ± 0.10 | <0.05 | <0.05 | <0.05 |
| Lung | 0.43 ± 0.15 | 0.11 ± 0.01 | 0.07 ± 0.02 | <0.05 |
| Spleen | 0.50 ± 0.09 | 0.10 ± 0.03 | <0.05 | <0.05 |
| Kidneys | 30 ± 2 | 11 ± 3 | 2.6 ± 0.7 | 0.63 ± 0.12 |
| Stomach | 0.41 ± 0.29 | 0.057 ± 0.004 | <0.05 | <0.05 |
| Intestines | 0.22 ± 0.05 | <0.05 | <0.05 | <0.05 |
| Liver | 0.14 ± 0.05 | <0.05 | <0.05 | <0.05 |
| Salivary glands | 0.27 ± 0.04 | 0.06 ± 0.01 | <0.05 | <0.05 |
| Muscle | 0.09 ± 0.03 | <0.05 | <0.05 | <0.05 |
| Bone | 0.14 ± 0.05 | <0.05 | <0.05 | <0.05 |

**3. Ex vivo SPECT imaging and autoradiography**

***Purpose:*** Ex vivo studies were performed following the collection of kidneys from mice injected with [^177^Lu]Lu-SibuDAB or [^177^Lu]Lu-PSMA-I&T to confirm the suborgan uptake pattern which is commonly seen for radiopharmaceuticals. In addition, the effect of excess unlabeled ligand on the uptake pattern of the radioligands in the kidneys was investigated.

***Method:*** Immunocompetent FVB mice were injected with [^177^Lu]Lu-SibuDAB or [^177^Lu]Lu-PSMA-I&T (20 MBq, 1 nmol per mouse, n=2 for each radioligand) and sacrificed 4 hours later. Additional mice (n=1 for each radioligand) were injected with 50-fold excess of respective unlabeled ligand (SibuDAB or PSMA-I&T, 50 nmol per mouse) 15 min prior to the radioligands. The kidneys were collected, weighed and the accumulated activity was measured using a γ-counter (Wallac Wizard 1480, PerkinElmer, Waltham MA, USA. One of the kidneys was scanned using SPECT/CT (NanoSPECT/CT, Mediso Medical Imaging Systems, Budapest, Hungary) for about 4 h. Images were prepared using VivoQuant post-processing software (version 3.5, inviCRO Imaging Services and Software, Boston, MA, USA). The second kidney of the same mouse was frozen in Tissue-Tek, sectioned using a cryotome (Cryostar NX70 Cryostat, Epredia, Kalamazoo, MI, USA) to obtain 10-μm sections which were then exposed to a phosphor screen (Super resolution screen Cat. No. 7001487, PerkinElmer, Waltham MA, USA) and read in a phosphor imager (Cyclone Plus, PerkinElmer, Waltham MA, USA) to obtain autoradiograms.

***Results:*** The results of the ex vivo autoradiography and SPECT imaging studies demonstrated that the activity was, indeed, accumulated in the renal cortex. The kidney uptake of [^177^Lu]Lu-PSMA-I&T at 4 h after injection was 14 ± 2% IA/g, which was reduced to about 2.9% IA/g when an excess of 50 nmol PSMA-I&T was pre-injected. The renal accumulation of [^177^Lu]Lu-SibuDAB at 4 h p.i. was 9.4 ± 0.2% IA/g, while 7.8% IA/g was still measured in the kidneys of the mouse pre-injected with an excess of 50 nmol unlabeled SibuDAB. These results imply that PSMA is expressed in the mouse kidneys, however, an additional mechanism is likely involved in the renal retention of [^177^Lu]Lu-SibuDAB beyond PSMA-mediated binding.

**4. Study I: Tolerability of [^161^Tb]Tb-SibuDAB compared to [^177^Lu]Lu-SibuDAB, [^161^Tb]Tb-PSMA-I&T and [^177^Lu]Lu-PSMA-I&T (30 MBq/mouse).**

The number of mice assigned to each group was estimated based on the results of a similar study, previously performed to assess the tolerability of ^161^Tb- and ^177^Lu-based somatostatin analogues published by Busslinger & Mapanao et al. [6]. The changes in leukocytes, erythrocytes and thrombocytes counts (percentage reduction and SD) on Day 10 after radiopeptide administration were used to calculate the “effect sizes” as input values for G*power software (version 3.1.9.7) [7]. The sample size was then obtained by setting the “alpha error probability” to 5% and the “power” to 95%.

The tolerability of 30 MBq [^161^Tb]Tb-SibuDAB (n=6) was assessed in mice and compared to that of 30 MBq [^177^Lu]Lu-SibuDAB (n=5), as well as to [^161^Tb]Tb-PSMA-I&T (n=6) and [^177^Lu]Lu-PSMA-I&T (n=6). This activity corresponds to ~1.2 GBq/kg in mice and was chosen as it would translate in ~6.8 GBq for human patients based on the ratio of the body mass between a 25-g mouse and a 70-kg human patient (70/0.025), in combination with a correction factor for species-specific surface area differences (1/12.3) [4]. Radioligands were diluted in saline (0.9% NaCl) containing 0.05% bovine serum albumin. From the 6 mice initially injected with 30 MBq [^177^Lu]Lu-SibuDAB, one mouse had to be euthanized on Day 10 after radioligand application due to body mass loss exceeding 15%, in accordance with the predefined endpoint criteria. Except for the relative body mass, all data from this mouse were excluded from the analyses. No critical histopathological were determined for this mouse.

**4.1. Body masses**

***Purpose:*** The body mass of mice was monitored as relevant parameter for the general health status of the mice over the entire study.

***Methods:*** The body mass was measured three times a week and expressed relative to the body mass determined on the day of injection (Day 0). Endpoint criteria were determined based on loss of body mass, behavior and signs of unease or pain using a pre-defined scoring system (e.g. ≥15% relative loss in body mass).

***Results:*** The results are reported mainly in the main article. The body mass of mice increased continuously over the course of the study in all groups of mice (Fig. S2).

**Fig. S2 a–f** Relative body masses of mice injected with 30 MBq (1 nmol) radioligand. **a** Relative body masses presented as average ± SD (represented by dotted lines) of the groups in comparison to control mice; **b–f** Relative body masses presented for individual mice and as the average presented in color; **b** Vehicle; **c** [^161^Tb]Tb-SibuDAB; **d** [^177^Lu]Lu-SibuDAB; **e** [^161^Tb]Tb-PSMA-I&T; **f** [^177^Lu]Lu-PSMA-I&T. The dotted and dashed lines indicate 5% and 10% body mass loss relative to the initial body mass, respectively

**4.2. Hematological changes**

***Purpose:*** Blood cell counts were determined on Day 10, Day 28 and at study end on Day 56 while blood smears were assessed at study end for each mouse in order to investigate whether the treatment of mice was well tolerated.

***Methods:*** Blood cell counts were determined on Days 10, 28 and 56 after injection using a veterinary hematology analyzer (VetScan HM5, Abaxis, Union City, CA, USA), as previously described [6]. Blood cell counts were analyzed using two-way ANOVA. The Dunnett’s multiple comparison post-test was applied to compare the results of treated groups to that of the control group. The Tukey’s multiple comparison post-test was employed to compare the outcome among the different groups of mice treated with the radioligands (GraphPad Prism, version 10.5.0). A *p-*value <0.05 was considered statistically significant.

***Results:*** The results are mainly described in the article and reported in Table S3.

**Table S3** Blood cell counts of mice assessed on Days 10, 28 and 56 after treatment with 30 MBq [^161^Tb]Tb-SibuDAB, [^177^Lu]Lu-SibuDAB, [^161^Tb]Tb-PSMA-I&T or [^177^Lu]Lu-PSMA-I&T

|  | **Control** | **[^161^Tb]Tb-SibuDAB** | **[^177^Lu]Lu-SibuDAB** | **[^161^Tb]Tb-PSMA-I&T** | **[^177^Lu]Lu-PSMA-I&T** |
| --- | --- | --- | --- | --- | --- |
|  | - | 30 MBq  (1 nmol) | 30 MBq  (1 nmol) | 30 MBq  (1 nmol) | 30 MBq  (1 nmol) |
|  | n=6 | n=6 | n=5 | n=6 | n=6 |
| **Day 10** |  |  |  |  |  |
| Leukocytes [10^9^ cells/L] | 6.7 ± 1.0 | 6.5 ± 2.1 | 6.9 ± 2.4 | 8.5 ± 1.6 | 6.7 ± 1.2 |
| Erythrocytes [10^12^ cells/L] | 10.0 ± 0.1 | 9.9 ± 0.2 | 9.9 ± 0.2 | 10.0 ± 0.2 | 9.9 ± 0.2 |
| Thrombocytes [10^9^ cells/L] | 535 ± 20 | 414 ± 68* | 387 ± 97* | 502 ± 16 | 503 ± 72 |
| **Day 28** |  |  |  |  |  |
| Leukocytes [10^9^ cells/L] | 7.2 ± 1.3 | 8.0 ± 0.5 | 6.8 ± 1.2 | 9.2 ± 3.7 | 6.3 ± 1.1 |
| Erythrocytes [10^12^ cells/L] | 10.1 ± 0.1 | 10.3 ± 0.2 | 10.2 ± 0.5 | 10.3 ± 0.5 | 10.0 ± 0.4 |
| Thrombocytes [10^9^ cells/L] | 559 ± 29 | 503 ± 55 | 550 ± 72 | 574 ± 66 | 582 ± 43 |
| **Day 56** |  |  |  |  |  |
| Leukocytes [10^9^ cells/L] | 5.2 ± 1.1 | 5.7 ± 1.9 | 8.0 ± 1.8 | 7.8 ± 3.9 | 6.5 ± 1.1 |
| Erythrocytes [10^12^ cells/L] | 10.0 ± 0.3 | 10.3 ± 0.3 | 10.0 ± 0.3 | 10.4 ± 0.4 | 9.9 ± 0.2 |
| Thrombocytes [10^9^ cells/L] | 583 ± 19 | 529 ± 48 | 476 ± 90* | 579 ± 76 | 582 ± 63 |

*Value significantly differed from that of the control group (*p*<0.05)

**4.3. Blood plasma chemistry**

***Purpose:*** Several blood plasma parameters were measured at the end of the study on Day 56 to evaluate potential signs of liver and kidney damage.

***Methods:*** Blood plasma parameters were determined at study end on Day 56 using blood plasma obtained from centrifuged retrobulbar blood samples collected in lithium-heparin tubes (Microvette, 200 LH, Sarstedt, Germany). The concentrations of blood urea nitrogen, alkaline phosphatase and albumin were determined using a dry chemistry analyzer (DRI-CHEM 4000i, FUJIFILM, Japan) as previously reported [8]. Blood plasma parameters were analyzed using one-way ANOVA. The Dunnett’s multiple comparison post-test was applied to compare the results of treated groups to that of the control group. The Tukey’s multiple comparison post-test was employed to compare the outcome among the different groups of mice treated with the radioligands (GraphPad Prism, version 10.5.0). A *p-*value <0.05 was considered statistically significant.

***Results:*** The results are reported mainly in the main article and listed in Table S4.

**Table S4** Blood plasma parameters measured on Day 56 after treatment of mice with 30 MBq [^161^Tb]Tb-SibuDAB, [^177^Lu]Lu-SibuDAB, [^161^Tb]Tb-PSMA-I&T or [^177^Lu]Lu-PSMA-I&T

|  | **Control** | **[^161^Tb]Tb-SibuDAB** | **[^177^Lu]Lu-SibuDAB** | **[^161^Tb]Tb-**  **PSMA-I&T** | **[^177^Lu]Lu-**  **PSMA-I&T** |
| --- | --- | --- | --- | --- | --- |
|  | - | 30 MBq  (1 nmol) | 30 MBq  (1 nmol) | 30 MBq  (1 nmol) | 30 MBq  (1 nmol) |
|  | n=6 | n=6 | n=5 | n=6 | n=6 |
| BUN [mM] | 12 ± 3 | 9.5 ± 1.6 | 9.7 ± 2.1 | 11 ± 2 | 11 ± 2 |
| ALP [U/L] | 86 ± 13 | 80 ± 20 | 88 ± 11 | 88 ± 8 | 89 ± 11 |
| ALB [g/L] | 22 ± 1 | 23 ± 2 | 21 ± 1 | 21 ± 2 | 21 ± 3 |

BUN = blood urea nitrogen; ALP = alkaline phosphatase; ALB = albumin. No value was significantly different from that of the control group (*p*>0.05)

**4.4. Organ masses and respective organ-to-brain mass ratios**

***Purpose:*** At study end, potential changes in the organ masses and organ-to-brain mass ratios of mice treated with one of the respective radioligands were assessed in comparison to untreated control mice.

***Methods:*** Immediately after euthanasia at study end on Day 56, the brain, kidneys, liver and spleen were collected and weighed to calculate the respective organ-to-brain mass ratios. Organ masses and corresponding organ mass-to-brain mass ratios were analyzed using one-way ANOVA. The Dunnett’s multiple comparison post-test was applied to compare the results of treated groups to that of the control group. The Tukey’s multiple comparison post-test was employed to compare the outcome of the different groups of mice treated with the radioligands (GraphPad Prism, version 10.5.0). A *p-*value <0.05 was considered statistically significant.

***Results:*** Organ masses of treated mice did not significantly differ from those of untreated controls, and similar among the different treatment groups. The same held true for the respective organ-to-brain mass ratios (Table S5).

**Table S5** Organ masses of mice measured on Day 56 after treatment of mice with 30 MBq [^161^Tb]Tb-SibuDAB, [^177^Lu]Lu-SibuDAB, [^161^Tb]Tb-PSMA-I&T or [^177^Lu]Lu-PSMA-I&T

|  | **Control** | **[^161^Tb]Tb-SibuDAB** | **[^177^Lu]Lu-SibuDAB** | **[^161^Tb]Tb-**  **PSMA-I&T** | **[^177^Lu]Lu-**  **PSMA-I&T** |
| --- | --- | --- | --- | --- | --- |
|  | - | 30 MBq  (1 nmol) | 30 MBq  (1 nmol) | 30 MBq  (1 nmol) | 30 MBq  (1 nmol) |
|  | n=6 | n=6 | n=5 | n=6 | n=6 |
| Body mass at euthanasia [g] | 26 ± 2 | 27 ± 2 | 26 ± 2 | 24 ± 1 | 26 ± 2 |
| Brain mass [mg] | 449 ± 31 | 476 ± 22 | 448 ± 17 | 425 ± 25 | 447 ± 26 |
| Kidney mass [mg] | 279 ± 17 | 312 ± 37 | 286 ± 13 | 276 ± 21 | 279 ± 20 |
| Liver mass [mg] | 1245 ± 166 | 1366 ± 199 | 1225 ± 62 | 1182 ± 124 | 1238 ± 155 |
| Spleen mass [mg] | 102 ± 13 | 109 ± 15 | 101 ± 8 | 91 ± 7 | 101 ± 11 |
| Kidney-to-Brain | 0.62 ± 0.04 | 0.66 ± 0.06 | 0.64 ± 0.05 | 0.65 ± 0.06 | 0.62 ± 0.03 |
| Liver-to-Brain | 2.8 ± 0.5 | 2.9 ± 0.3 | 2.7 ± 0.2 | 2.8 ± 0.3 | 2.8 ± 0.2 |
| Spleen-to-Brain | 0.23 ± 0.04 | 0.23 ± 0.03 | 0.23 ± 0.02 | 0.22 ± 0.02 | 0.23 ± 0.01 |

No value was significantly different from that of the control group (*p*>0.05)

**4.5. Histopathological analyses of the blood and bone marrow smears**

***Purpose:*** The effects of the radioligands on the blood cells present in peripheral blood and the bone marrow were evaluated using respective smears to determine potential effects of the radioligands.

***Methods:*** At the end of the study on Day 56, blood smears from sublingual blood and bone marrow smears were prepared and stained using the Pappenheim method [9]. Semi-quantitative analysis of the bone marrow cells included counts of at least 500 erythroid and myeloid cells. The myeloid-to-erythroid ratio (M:E) was assessed qualitatively without differentiating the maturation stages of each cell type. The presence or absence of cellular abnormalities was also recorded. Potential morphological changes of the blood cells were assessed by a board-certified veterinary pathologist (AnaPath Services GmbH, Liestal, Switzerland). Myeloid-to-erythroid ratios were analyzed using one-way ANOVA. The Dunnett’s multiple comparison post-test was applied to compare the results of treated groups to that of the control group. The Tukey’s multiple comparison post-test was employed to compare the values among the different groups of mice treated with the radioligands (GraphPad Prism, version 10.5.0). A *p-*value <0.05 was considered statistically significant.

***Results:*** The results are described in the main article. Summary of the findings on the blood smears are reported in Table S6. The myeloid-to-erythroid ratio based on the analyses of bone marrow smears are reported in Table S7.

**Table S6** Overview of the assessment of blood smears on Day 56 of mice that received 30 MBq [^161^Tb]Tb-SibuDAB, [^177^Lu]Lu-SibuDAB, [^161^Tb]Tb-PSMA-I&T or [^177^Lu]Lu-PSMA-I&T in comparison to findings made for mice of the control group

|  | **Control** | **[^161^Tb]Tb-SibuDAB** | **[^177^Lu]Lu-SibuDAB** | **[^161^Tb]Tb-**  **PSMA-I&T** | **[^177^Lu]Lu-**  **PSMA-I&T** |
| --- | --- | --- | --- | --- | --- |
|  | - | 30 MBq  (1 nmol) | 30 MBq  (1 nmol) | 30 MBq  (1 nmol) | 30 MBq  (1 nmol) |
|  | n=6 | n=6 | n=5 | n=6 | n=6 |
| Metamyelocytes | Present  (n=6) | Increased  (n=6) | Increased  (n=5) | Increased  (n=6) | Increased  (n=6) |
| Band neutrophils | Present  (n=2) | Increased  (n=5) | Increased  (n=3) | Increased  (n=4) | Increased  (n=3) |
| Hypersegmented neutrophils |  | Present  (n=1) | Present  (n=5) | Present  (n=6) | Present  (n=6) |
| Late myeloblast |  |  | Present  (n=2) | Present  (n=2) |  |
| Metarubricytes |  |  | Present  (n=2) |  | Present  (n=3) |
| Thrombocytes |  |  |  | Increased^#^  (n=1) |  |
| Reticulocytes |  |  |  | Decreased^#^  (n=1) |  |

^#^Relative to that in control mice

**Table S7** Overview of the assessment of blood smears on Day 56 of mice that received 30 MBq [^161^Tb]Tb-SibuDAB, [^177^Lu]Lu-SibuDAB, [^161^Tb]Tb-PSMA-I&T or [^177^Lu]Lu-PSMA-I&T in comparison to findings made for mice of the control group

|  | **Control** | **[^161^Tb]Tb-SibuDAB** | **[^177^Lu]Lu-SibuDAB** | **[^161^Tb]Tb-**  **PSMA-I&T** | **[^177^Lu]Lu-**  **PSMA-I&T** |
| --- | --- | --- | --- | --- | --- |
|  | - | 30 MBq  (1 nmol) | 30 MBq  (1 nmol) | 30 MBq  (1 nmol) | 30 MBq  (1 nmol) |
|  | n=6 | n=6 | n=5 | n=6 | n=6 |
| Myeloid-to-Erythroid Ratio | 1.22 ± 0.37 | 1.64 ± 0.63 | 1.51 ± 0.30 | 1.86 ± 0.81 | 1.75 ± 0.52 |

No value was significantly different from that of the control group (*p*>0.05)

**4.6. Histopathological analyses of the spleen, liver and kidneys**

***Purpose:*** The occurrence of potential early side effects in spleen, liver and kidneys after treatment with 30 MBq [^161^Tb]Tb-SibuDAB, [^177^Lu]Lu-SibuDAB, [^161^Tb]Tb-PSMA-I&T or [^177^Lu]Lu-PSMA-I&T were assessed based on histo(patho)logical analysis of the respective tissue sections.

***Methods:*** After organ collection and weighing, the spleen, liver and kidneys were fixed in neutral-buffered formaldehyde (4%) and embedded in paraffin. Tissue sections of 4-μm thickness were stained with hematoxylin and eosin and images were taken under a light microscope (Olympus CX23, Evident Corporation, Nagano, Japan) for analysis performed by board-certified veterinary pathologist (AnaPath Services GmbH, Liestal, Switzerland), following a pre-defined scoring system (Table S8) [6].

**Table S8** Score system for histopathological analysis of selected tissues

| **Score** | **Definition** |
| --- | --- |
| Score 1  Minimal | Minor, small or infrequent histopathologic changes ranging from inconspicuous to barely noticeable. For multifocal or diffusely distributed lesions, this score was used for processes in which less than 10% of the tissue in an average high-power field was involved. |
| Score 2  Slight | Histopathologic changes that were noticeable but not a prominent feature of the tissue. For multifocal or diffusely distributed lesions, this score was used for processes in which 10% to 25% of the tissue in an average high-power field were involved. |
| Score 3  Moderate | Histopathologic changes that were prominent but not dominant features of the tissue. For multifocal or diffusely distributed lesions, this score was used for processes in which 25% to 50% of the tissue in an average high-power field were involved. |
| Score 4  Marked | Histopathologic changes that were dominant, but not an overwhelming feature of the tissue. For multifocal or diffusely distributed lesions, this score was used for processes in which 50% to 95% of the tissue in an average high-power field was involved. |
| Score 5  Severe | Histopathologic changes that were an overwhelming feature of the tissue. For multifocal or diffusely distributed lesions, this score was used for processes in which more than 95% of the tissue in an average high-power field was involved. |

***Results:*** The results are described in the main article, presented in Fig. S3.

**Fig. S3 a–d** Summary of the most relevant histopathological findings in the spleen, liver and kidney of mice injected with the respective radioligand (30 MBq, 1 nmol per mouse) in comparison to healthy control mice. Severity scores were assigned to each mouse of each group. **a** Splenic lymphoid hyperplasia, **b** hepatocellular hypertrophy, **c** renal tubular degeneration and **d** renal tubular basophilia. Bars indicate the average score for each treatment group

**5. Study II: Tolerability of [^161^Tb]Tb-SibuDAB at increasing activities (15, 30 and 60 MBq/mouse)**

Activity-dependent effects were investigated in mice injected with 15 MBq (n=6), 30 MBq (n=6) (same group as included in Study I) and 60 MBq of [^161^Tb]Tb-SibuDAB (n=6). Based on the abovementioned scaling approach, the administered mouse activities would correspond to approximately 3.4 GBq, 6.8 GBq and 13.7 GBq in humans, respectively.

**5.1. Body masses**

***Purpose:*** The body mass of mice was monitored as relevant parameter for the general health status of the mice over the entire study.

***Methods:*** Measurement of the body masses and calculation of the relative body mass of mice injected with 15 MBq, 30 MBq or 60 MBq of [^161^Tb]Tb-SibuDAB were determined as reported for Study I.

***Results:*** The results are reported mainly in the main article. The average body mass of all mice over time are shown in Fig. S4.

**Fig. S4 a–e** Relative body masses of mice injected with [^161^Tb]Tb-SibuDAB at variable activities. **a** Comparison of all groups shown as average ± SD (shown as dotted lines); **b–e** Relative body masses presented for individual mice and as the average presented in color. **b** Vehicle; **c** [^161^Tb]Tb-SibuDAB (15 MBq, 1 nmol per mouse); **d** [^161^Tb]Tb-SibuDAB (30 MBq, 1 nmol per mouse); **e** [^161^Tb]Tb-SibuDAB (60 MBq, 1 nmol per mouse). The dotted and dashed lines indicate 5% and 10% body mass loss relative to the initial body mass, respectively

**5.2. Hematological changes**

***Purpose:*** The influence of the radioligands on blood cell counts was assessed on Days 10, 28 and 56 after injection of the mice.

***Methods:*** Blood cell counts were determined and analyzed as reported for Study I.

***Results:*** The results are reported mainly in the main article and the determined blood cell counts listed in Table S9.

**Table S9** Blood cell counts of mice assessed on Day 10, Day 28 and Day 56 after treatment with 15 MBq, 30 MBq or 60 MBq [^161^Tb]Tb-SibuDAB

|  | **Control** | **[^161^Tb]Tb-SibuDAB** | | |
| --- | --- | --- | --- | --- |
|  | - | 15 MBq  (1 nmol) | 30 MBq  (1 nmol) | 60 MBq  (1 nmol) |
|  | n=6 | n=6 | n=6 | n=6 |
| **Day 10** |  |  |  |  |
| Leukocytes [10^9^ cells/L] | 6.7 ± 1.0 | 5.8 ± 0.4 | 6.5 ± 2.1 | 5.1 ± 1.0 |
| Erythrocytes [10^12^ cells/L] | 10.0 ± 0.1 | 10.1 ± 0.2 | 9.9 ± 0.2 | 9.3 ± 0.3* |
| Thrombocytes [10^9^ cells/L] | 535 ± 20 | 438 ± 36* | 414 ± 68* | 300 ± 76* |
| **Day 28** |  |  |  |  |
| Leukocytes [10^9^ cells/L] | 7.2 ± 1.3 | 7.5 ± 1.7 | 8.0 ± 0.5 | 5.8 ± 1.3 |
| Erythrocytes [10^12^ cells/L] | 10.1 ± 0.1 | 10.2 ± 0.1 | 10.3 ± 0.2 | 9.8 ± 0.2* |
| Thrombocytes [10^9^ cells/L] | 559 ± 29 | 483 ± 28 | 503 ± 55 | 403 ± 85* |
| **Day 56** |  |  |  |  |
| Leukocytes [10^9^ cells/L] | 5.2 ± 1.1 | 7.7 ± 1.8* | 5.7 ± 1.9 | 6.0 ± 1.2 |
| Erythrocytes [10^12^ cells/L] | 10.0 ± 0.3 | 10.3 ± 0.4 | 10.3 ± 0.3 | 10.0 ± 0.1 |
| Thrombocytes [10^9^ cells/L] | 583 ± 19 | 446 ± 133* | 529 ± 48 | 478 ± 54* |

*Value significantly differed from that of the control group (*p*<0.05)

**5.3 Blood plasma chemistry**

***Purpose:*** Several blood plasma parameters were measured at study end to assess the mice for potential signs of kidney and liver damage.

***Methods:*** Blood plasma parameters were determined and analyzed as reported for Study I.

***Results:*** The results are reported mainly in the main article and listed in Table S10.

**Table S10** Blood plasma parameters measured on Day 56 after treatment of mice with 15 MBq, 30 MBq or 60 MBq [^161^Tb]Tb-SibuDAB

|  | **Control** | **[^161^Tb]Tb-SibuDAB** | | | |
| --- | --- | --- | --- | --- | --- |
|  | - | 15 MBq  (1 nmol) | 30 MBq  (1 nmol) | 60 MBq  (1 nmol) |  |
|  | n=6 | n=6 | n=6 | n=6 |  |
| BUN [mM] | 12 ± 3 | 9.7 ± 1.8 | 9.5 ± 1.6 | 8.4 ± 1.6* |  |
| ALP [U/L] | 86 ± 13 | 80 ± 7 | 80 ± 20 | 90 ± 5 |  |
| ALB [g/L] | 22 ± 1 | 24 ± 2 | 23 ± 2 | 22 ± 2 |  |

BUN = blood urea nitrogen; ALP = alkaline phosphatase; ALB = albumin. *Value significantly differed from that of the control group (*p*<0.05)

**5.4. Organ masses and respective organ-to-brain mass ratios**

***Purpose:*** At study end, potential changes in the organ masses and organ-to-brain mass ratios of mice treated with one of the respective radioligands were assessed in comparison to untreated control mice.

***Methods*** The organ masses and respective organ-to-brain mass ratios were assessed and analyzed as reported for Study I.

***Results:*** The results are reported mainly in the main article and listed in Table S11.

**Table S11** Organ masses of mice measured on Day 56 after treatment of mice with 15 MBq, 30 MBq or 60 MBq [^161^Tb]Tb-SibuDAB

|  | **Control** | **[^161^Tb]Tb-SibuDAB** | | | |
| --- | --- | --- | --- | --- | --- |
|  | - | 15 MBq  (1 nmol) | 30 MBq  (1 nmol) | 60 MBq  (1 nmol) |  |
|  | n=6 | n=6 | n=6 | n=6 |  |
| Body mass at euthanasia [g] | 26 ± 2 | 24 ± 2 | 27 ± 2 | 26 ± 1 |  |
| Brain mass [mg] | 449 ± 31 | 444 ± 11 | 476 ± 22 | 449 ± 18 |  |
| Kidney mass [mg] | 279 ± 17 | 271 ± 15 | 312 ± 37 | 322 ± 32* |  |
| Liver mass [mg] | 1245 ± 166 | 1147 ± 117 | 1366 ± 199 | 1201 ± 131 |  |
| Spleen mass [mg] | 102 ± 13 | 97 ± 9 | 109 ± 15 | 102 ± 14 |  |
| Kidney-to-Brain | 0.62 ± 0.04 | 0.61 ± 0.03 | 0.66 ± 0.06 | 0.72 ± 0.07* |  |
| Liver-to-Brain | 2.8 ± 0.5 | 2.6 ± 0.3 | 2.9 ± 0.3 | 2.7 ± 0.3 |  |
| Spleen-to-Brain | 0.23 ± 0.04 | 0.22 ± 0.02 | 0.23 ± 0.03 | 0.23 ± 0.03 |  |

*Value significantly differed from that of the control group (*p*<0.05).

**5.5. Histopathological analyses of the blood and bone marrow smears**

***Purpose:*** The effects of the radioligands on the blood cells present in peripheral blood and the bone marrow were evaluated using respective smears to determine potential effects of the radioligands.

***Methods:*** The blood and bone marrow smears were prepared, stained and analyzed, as described in Study I.

***Results:*** The results are mainly discussed in the main article. Summary of the findings on blood smears are reported in Table S12. The myeloid-to-erythroid ratio based on the analyses of bone marrow smears are reported in Table S13.

**Table S12** Overview of the assessment of blood smears on Day 56 of mice that received 15 MBq, 30 MBq or 60 MBq [^161^Tb]Tb-SibuDAB in comparison to findings made for mice of the control group

|  | **Control** | **[^161^Tb]Tb-SibuDAB** | | |
| --- | --- | --- | --- | --- |
|  | - | 15 MBq  (1 nmol) | 30 MBq  (1 nmol) | 60 MBq  (1 nmol) |
|  | n=6 | n=6 | n=6 | n=6 |
| Metamyelocytes | Present  (n=6) | Increased  (n=6) | Increased  (n=6) | Increased  (n=5) |
| Band neutrophils | Present  (n=2) | Increased  (n=5) | Increased  (n=5) | Present  (n=1)  Increased  (n=2) |
| Hypersegmented neutrophils |  | Present  (n=6) | Present  (n=1) | Present  (n=3) |
| Late myeloblast |  | Present  (n=3) |  |  |

**Table S13** Overview of the assessment of blood smears on Day 56 of mice that received 15 MBq, 30 MBq or 60 MBq [^161^Tb]Tb-SibuDAB in comparison to findings made for mice of the control group

|  | **Control** | **[^161^Tb]Tb-SibuDAB** | | |
| --- | --- | --- | --- | --- |
|  | - | 15 MBq  (1 nmol) | 30 MBq  (1 nmol) | 60 MBq  (1 nmol) |
|  | n=6 | n=6 | n=6 | n=6 |
| Myeloid-to-Erythroid Ratio | 1.22 ± 0.37 | 1.39 ± 0.41 | 1.64 ± 0.63 | 1.43 ± 0.50 |

No value was significantly different from that of the control group (*p*>0.05)

**5.6. Histopathological analyses of the spleen, liver and kidneys**

***Purpose:*** The occurrence of potential early side effects in spleen, kidneys and liver after treatment with 15 MBq, 30 MBq or 60 MBq [^161^Tb]Tb-SibuDAB, were assessed based on histo(patho)logical analysis of the respective tissue sections.

***Methods:*** Histopathological investigations were performed and analyzed as described for Study I.

***Results:*** The results are described in the main article. A summary of the most relevant histopathological findings are shown in Fig. S5.

**Fig. S5 a–d** Summary of the most relevant histopathological findings in the spleen, liver and kidneys of mice injected with 15 MBq, 30 MBq or 60 MBq [^161^Tb]Tb-SibuDAB. Severity scores were assigned to each mouse of each group. **a** Splenic lymphoid hyperplasia, **b** hepatocellular hypertrophy, **c** renal tubular degeneration and **d** renal tubular basophilia. Bars indicate the average score for each treatment group

**References**

1.	Borgna F, Deberle LM, Busslinger SD, Tschan VJ, Walde LM, Becker AE, et al. Preclinical investigations to explore the difference between the diastereomers [^177^Lu]Lu-SibuDAB and [^177^Lu]Lu-RibuDAB toward prostate cancer therapy. Mol Pharm. 2022;19:2105-14. doi:10.1021/acs.molpharmaceut.1c00994.

2.	Gracheva N, Müller C, Talip Z, Heinitz S, Köster U, Zeevaart JR, et al. Production and characterization of no-carrier-added ^161^Tb as an alternative to the clinically-applied ^177^Lu for radionuclide therapy. EJNMMI Radiopharm Chem. 2019;4:12. doi:10.1186/s41181-019-0063-6.

3.	Tschan VJ, Busslinger SD, Bernhardt P, Grundler PV, Zeevaart JR, Köster U, et al. Albumin-binding and conventional PSMA ligands in combination with ^161^Tb: biodistribution, dosimetry, and preclinical therapy. J Nucl Med. 2023;64:1625-31. doi:10.2967/jnumed.123.265524.

4.	Nair AB, Jacob S. A simple practice guide for dose conversion between animals and human. J Basic Clin Pharm. 2016;7:27-31. doi:10.4103/0976-0105.177703.

5.	Müller C, Umbricht CA, Gracheva N, Tschan VJ, Pellegrini G, Bernhardt P, et al. Terbium-161 for PSMA-targeted radionuclide therapy of prostate cancer. Eur J Nucl Med Mol Imaging. 2019;46:1919-30. doi:10.1007/s00259-019-04345-0.

6.	Busslinger SD, Mapanao AK, Kegler K, Bernhardt P, Flühmann F, Fricke J, et al. Comparison of the tolerability of ^161^Tb- and ^177^Lu-labeled somatostatin analogues in the preclinical setting. Eur J Nucl Med Mol Imaging. 2024;51:4049-61. doi:10.1007/s00259-024-06827-2.

7.	Faul F, Erdfelder E, Buchner A, Lang AG. Statistical power analyses using G*Power 3.1: tests for correlation and regression analyses. Behav Res Methods. 2009;41:1149-60. doi:10.3758/BRM.41.4.1149.

8.	Tschan VJ, Borgna F, Busslinger SD, Stirn M, Rodriguez JMM, Bernhardt P, et al. Preclinical investigations using [^177^Lu]Lu-Ibu-DAB-PSMA toward its clinical translation for radioligand therapy of prostate cancer. Eur J Nucl Med Mol Imaging. 2022;49:3639-50. doi:10.1007/s00259-022-05837-2.

9.	Binder T, Diem H, Fuchs R, Gutensohn K, Nebe T. Pappenheim stain: description of a hematological standard stain - history, chemistry, procedure, artifacts and problem solutions. J Lab Med. 2012;36:293-309. doi:10.1515/labmed-2012-0027.
